# Staples, tension-band plates, and percutaneous epiphysiodesis screws used for leg-length discrepancy treatment: a systematic review and proportional meta-analysis

**DOI:** 10.2340/17453674.2024.41104

**Published:** 2024-07-18

**Authors:** Maria TIRTA, Mette Holm HJORTH, Jette Frost JEPSEN, Søren KOLD, Ole RAHBEK

**Affiliations:** 1 Interdisciplinary Orthopedics, Aalborg University Hospital; 2 Department of Orthopedics Surgery, Aarhus University Hospital; 3 Medical Library, Aalborg University Hospital, Denmark

## Abstract

**Background and purpose:**

The primary aim of this systematic review and meta-analysis was to evaluate the success rate of 3 different epiphysiodesis techniques with implant usage for the treatment of leg-length discrepancy (LLD) in the pediatric population. The secondary aim was to address effectiveness (final LLD) and the reported complications of staples, tension-band plates (TBP), and percutaneous epiphysiodesis screws (PETS).

**Methods:**

In this systematic review we searched MEDLINE (PubMed), Embase, Cochrane Library, Web of Science and Scopus for studies on skeletally immature patients with LLD treated with epiphysiodesis with an implant. The extracted outcome categories were effectiveness of epiphysiodesis (LLD measurements pre-/postoperatively, successful/unsuccessful) and complications that were graded on severity.

**Results:**

44 studies (2,184 patients) were included. 455 underwent epiphysiodesis with PETS, 578 patients with TBP, and 1,048 with staples. Successful epiphysiodesis was reported in 76% (95% confidence interval [CI] 61–89) with PETS (9 studies), 67% (CI 54–79) with TBP (10 studies), and 51% (CI 28–65) with Blount staples (8 studies). From pooled analysis, the severe complications rate was 7% for PETS, 17% for TBP, and 16% for Blount staples. Angular deformity was reported in 4% after PETS, 10% after TBP, and 17% after Blount staples.

**Conclusion:**

Our results showed that epiphysiodesis with PETS implants was the most successful technique. PETS had a higher success rate, fewer severe complications, and a lower proportion with angular deformity.

Epiphysiodesis, defined as the process of closing the growth plate (physis), has been used for several years as a treatment option in cases where the predicted leg-length discrepancy (LLD) falls between 2 and 5 cm [1].

Reversibility of epiphysiodesis was an aspect that Blount and Clarke [2] first attempted to use by positioning staples spanning the physis on both the medial and lateral side. However, staples have been linked with several complications, especially with hardware failure resulting in angular deformities [3]. In 2007, Stevens [4] introduced an alternative method for reversible epiphysiodesis, which involved utilizing an eight-plate along with 2 non-locked screws, also known as tension-band plates (TBP). TBP can function as a flexible tension band construct, meaning it does not apply immediate and direct compressive force but theoretically places the fulcrum outside the bone, giving different compressive force on the physis compared with staples [5]. In addition, percutaneous epiphysiodesis with transphyseal screws (PETS) has been used as another technique of guided growth because it imposes direct compressive stress across the physis and slows growth [6].

The efficiency in terms of growth arrest and success rate, as well as the complications of these techniques, remains controversial [7-8], and as a result a systematic review was indicated.

The primary aim of this systematic,review and meta-analysis was to evaluate the success rate of 3 different epiphysiodesis techniques with implant usage for the treatment of leg-length discrepancy (LLD) in the pediatric population. The secondary aim was to address effectiveness (final LLD) and the reported complications of staples, tension-band plates (TBP), and percutaneous epiphysiodesis screws (PETS).

## Methods

This systematic review and meta-analysis were performed according to Preferred Items for Systematic Reviews and Meta-Analyses (PRISMA) guidelines [9] and registered with PROSPERO (CRD42023465953).

### Information sources and search strategy

We identified eligible studies by conducting a systematic search of electronic databases using a predetermined search strategy. Our search encompassed MEDLINE (PubMed), Embase, the Cochrane Library, Web of Science, and Scopus (last search date May 25, 2023). The search was carried out using combinations of the terms “epiphysiodesis,” “limb length discrepancy,” “leg length discrepancy’’, “physiodesis,” “tension band plating,” “guided growth,’’ and “eight plate”. Table S1 (see Supplementary data) presents the search strategy. To avoid including redundant or duplicated samples, we meticulously compared all the studies. If any overlap was identified, we selected the study with the highest count of events for inclusion. We did not impose any limitations based on publication dates or language. Moreover, to guarantee comprehensive coverage of the available literature, we performed an extensive search of the reference lists of the studies that were included, relevant reviews identified through our systematic search, and the personal archives of the authors.

### Eligibility criteria

Inclusion criteria were as follows: (i) skeletally immature pediatric population, defined as growth being complete at age 14 years for girls and age 16 years for boys; (ii) LLD that was treated surgically; and (iii) use of 1 of the following epiphysiodesis techniques: staples, TBP or PETS, or any other epiphysiodesis technique with an implant.

Exclusion criteria were: (i) nonoperative treatment of LLD, or leg lengthening surgical treatment; (ii) epiphysiodesis for tall stature; (iii) hemiepiphysiodesis and angular deformity correction; and (iv) permanent epiphysiodesis treatment including Phemister and the modified Phemister technique, percutaneous epiphysiodesis (PE) with drills/curette, or any other percutaneous technique that was mentioned as permanent epiphysiodesis.

Considering the study’s design, randomized controlled trials (RCTs), controlled (non-randomized) clinical trials (CCTs), prospective and retrospective cohort studies, case-control or nested case-control studies, analytical cross-sectional studies, and case series were eligible for inclusion. Only human studies were included. We excluded case reports (defined as articles that describe and interpret 1–3 individual cases), letters, editorials, reviews, and commentaries.

### Intervention: surgical techniques information

PETS employs percutaneously introduced cannulated “lag screws” under fluoroscopy to achieve permanent physis compression, thereby inhibiting further growth through the physis. These lag screws are positioned on both the medial and lateral sides of the physis and can be oriented either in parallel or, more commonly, in a crossed configuration [6].

The Blount staples technique necessitates the placement of 3 staples per side around the physis, with each staple having 2 leg anchored in the metaphysis and the other in the epiphysis on both the medial and lateral sides [2]. Like staples, TBP are positioned on both the medial and lateral sides of the physis, with one portion firmly anchored in the metaphyseal bone and the other in the epiphyseal bone [5].

### Selection process

All studies identified through the search strategy were subjected to screening based on the eligibility criteria. The literature search was carried out independently by 2 authors (MT and JFJ), and any disagreements were resolved through discussions to establish a consensus. If consensus remained elusive, a third author was consulted for guidance.

### Data-collection process

The characteristics of each study included in the analysis were assessed using a predefined data extraction form outlined in the Cochrane Handbook for Systematic Reviews. 2 reviewers independently extracted the following information from each study: study details (first author, publication year, and country), study type, epiphysiodesis type, study duration, inclusion/exclusion criteria, preoperative assessments (skeletal maturity definition, timing of epiphysiodesis, predicted length at maturity), surgery details (technique, postoperative protocol), sample size, mean age, sex, etiology of limb length discrepancy (LLD), side and bone of epiphysiodesis, and follow-up duration.

The main outcome was considered effectiveness of epiphysiodesis by success rate (success/failure). Secondary outcomes were effectiveness by final LLD (LLD measurements pre-/postoperatively), physeal fusion/arrest or efficacy of the epiphysiodesis, and complications. Complications were classified using the system developed by Black et al. [10] (Table 1). MT identified and assessed all complications for severity, and a second reviewer (SK) independently evaluated and graded them. In instances of disagreement between reviewers, a consensus discussion was conducted to resolve discrepancies.

**Table 1:** Complication classification according to Black et al. [10]

Category	Definition	Examples
I	Minimal intervention required; treatment goal still achieved.	Infection treated by antibiotics, effusion/edema, hematoma/hemarthrosis, knee pain, reduced knee range of motion, wound dehiscence/healing, skin burn/skin blistering, peroneal nerve neuropathy.
II	Substantial change in treatment plan; treatment goal still achieved.	Further surgical intervention/reoperation, infection treated by debridement or revision, wound dehiscence/healing if additional surgery is required, skin burn/skin blistering if additional surgery is required.
IIIA	Failure to achieve treatment goal; no new pathology or permanent sequelae.	Fracture, failure of growth plate arrest, failure to achieve adequate reduction in LLD, additional surgeries needed for LLD treatmen.t
IIIB	Failure to achieve treatment goal and/or new pathology or permanent sequelae developed	Overcorrection, angular deformity, genu recurvatum, asymmetrical closure of the growth plate with progressive malalignment, exostosis, neurapraxia.

A proportional meta-analysis was conducted for the main outcome of success rate of each surgical technique. A systematic review was undertaken, presenting a combined analysis, and a narrative synthesis of the findings was performed for final LLD and complications.

### Quality assessment

2 reviewers conducted an independent assessment of the quality of the studies included. In cases where there was a discrepancy between the decisions made by the 2 reviewers, a third reviewer examined the content and made the determination regarding inclusion or exclusion. The methodological quality of the studies was evaluated using the Methodological Index for Non-Randomized Studies (MINORS) for non-randomized studies [11]. Each item in the MINORS criteria was scored as 0 (not reported), 1 (reported but inadequate), or 2 (reported and adequate). The maximum achievable score is 16 for non-comparative studies and 24 for comparative studies. For the MINORS criteria along with explanations for each item, see Supplementary data.

### Statistics

A meta-analysis of proportions was conducted for the available information on successful/unsuccessful LLD treatment (success rate) as the main outcome of effectiveness. Forest plots for all 3 methods were made to illustrate group-specific results. Inconsistency was assessed using I2, describing the percentage of total variation across the studies due to heterogeneity rather than chance (low inconsistency: I2 < 40%, moderate inconsistency: I2 between 30% and 60%, substantial inconsistency: I2 between 50% and 90%, large inconsistency: I2 > 75–100%). The meta-analysis was performed with a standard pairwise, using a random effect model, meta-analysis (REML). Sensitivity analysis was additionally performed.

The analyses were conducted in STATA 18.0 (StataCorp LLC, College Station, TX, USA).

### Ethics, data sharing, funding, use of AI, and disclosures

An ethics statement is not applicable because this study is based on published literature. The data that supports the findings of this study is available from the corresponding author upon reasonable request. There was no use of artificial intelligence (AI) for the production of the current study. There was no funding for this study. The authors declare no conflicts of interest. Complete disclosure of interest forms according to ICMJE are available on the article page, doi: 10.2340/17453674.2024.41104

## Results

### Data search results

The initial search across the specified databases produced a total of 1,469 articles. After eliminating duplicates and assessing titles and abstracts based on eligibility criteria, 59 articles were identified for a comprehensive review of their full texts. The screening process involved the application of both inclusion and exclusion criteria to determine suitability. 15 articles were excluded based on the screening process (Table S2, Supplementary data), leaving a total of 44 studies that were included in this systematic review.

**Figure 1:**
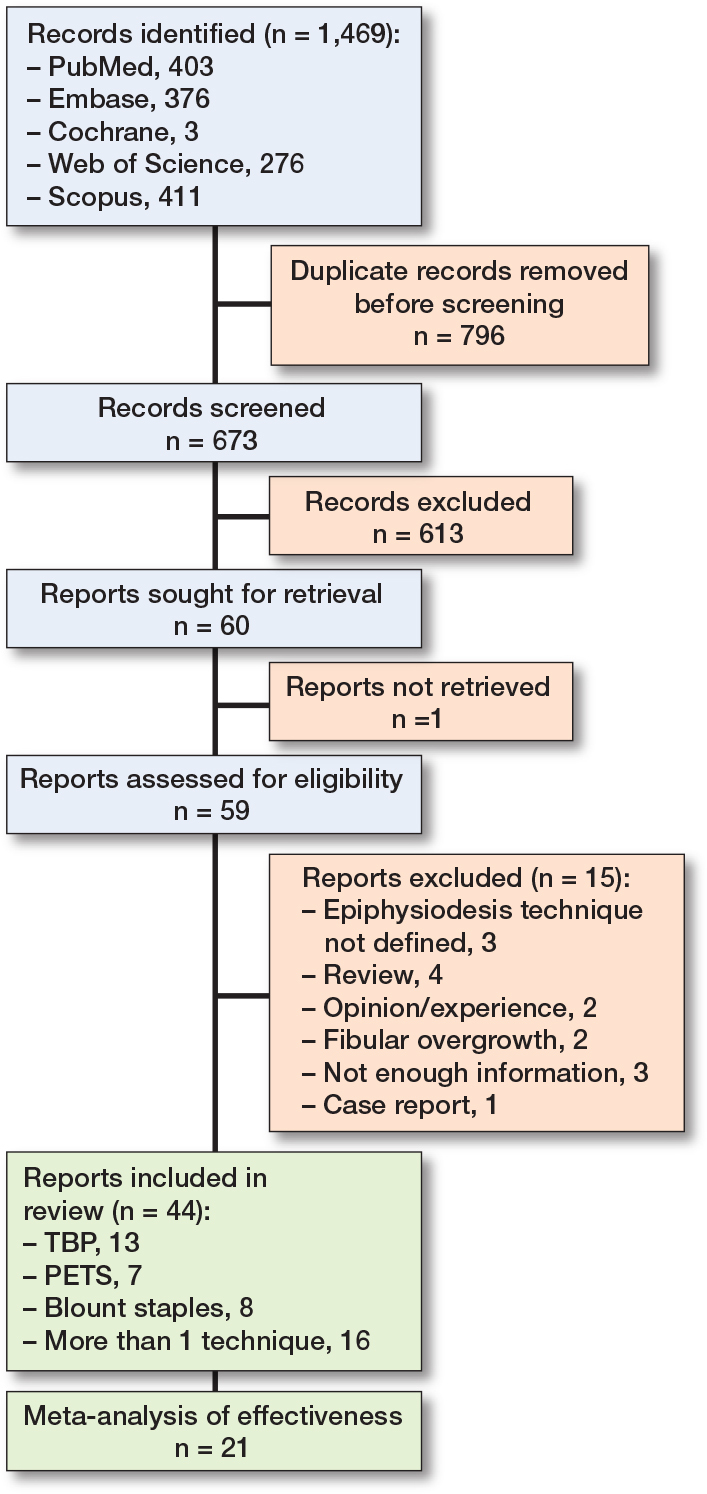
Flowchart of selection process for included studies.

Epiphysiodesis with TBP was presented in 13 studies [12-24], with PETS in 7 studies [6,25-30], and with Blount staples in 8 studies [3,31-37], while 16 studies included more than 1 surgical technique [7-8,38-51]. From the studies that included also permanent epiphysiodesis techniques (percutaneous epiphysiodesis, Phemister technique) we exclusively extracted and presented information considering staples, TBP, or PETS, or any other epiphysiodesis technique with an implant. 21 studies were included for the meta-analysis. The literature selection process, in accordance with the PRISMA guidelines, is depicted in Figure 1.

### Quality assessment of included studies

In non-comparative studies, the mean MINORS score was 8 (n = 32, ranging from 3–11, with an optimal score of 16). Comparative studies exhibited a mean MINORS score of 14 (n = 12, ranging from 12–17, with an optimal score of 24). A detailed breakdown of scores for each study can be found in Table S4 (see Supplementary data).

### Demographic characteristics

2,184 patients were included. 578 underwent epiphysiodesis with TBP, 455 with PETS, 1,048 with staples, and 103 with rigid staples. Most of the included studies were retrospective observational studies/case series/retrospective review, except for 1 cost analysis and 1 prospective study also with use of retrospective data. 32 studies were published between 2010 and 2022, 5 studies between 2000 and 2009, and 7 studies before 2000. 13 studies were conducted in the USA, 18 in Europe, and 3 in Turkey.

The preoperative assessment methods were reported in nearly all studies, excluding 4. The primary assessment tool was long standing radiographs, followed by scanograms. In 18 studies (41%), patients were monitored until maturity, while 8 studies did not specify the duration of follow-up. Various methods were employed to predict final leg length discrepancy (LLD), with the Paley multiplier method being the most utilized in 7 studies, followed by the Green–Anderson growth-remaining model in 3 studies. 21 studies (57%) did not provide details on the prediction method used. Table S3 (see Supplementary data) presents a comprehensive overview of each study’s characteristics.

### Effectiveness of TBP, PETS, and Blount staples: synthesis of success rate

**Figure 2:**
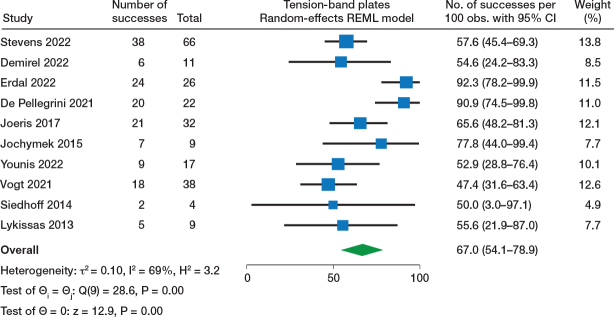
Forest plot of TBP (tension-band plates) and success rate. CI = confidence interval, τ^2^ = between-study variance, I^2^ = inconsistency.

**Figure 3:**
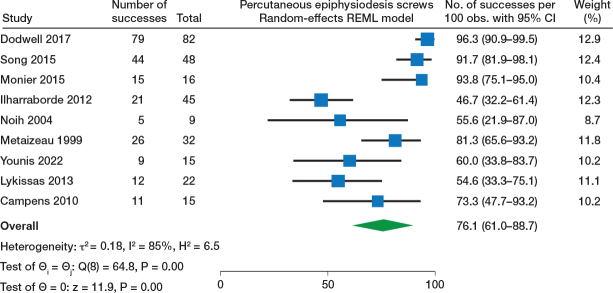
Forest plot of PETS (percutaneous epiphysiodesis screws) and success rate. For abbreviations, see Figure 2.

**Figure 4:**
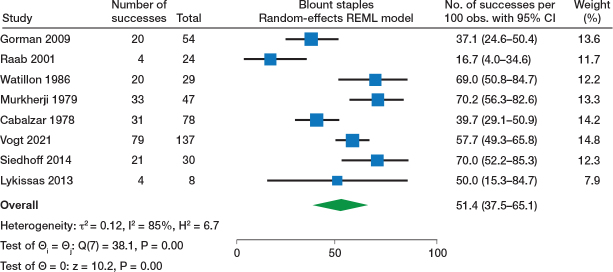
Forest plot of Blount staples and success rate. For abbreviations, see Figure 2.

Effectiveness of TBP (10 studies) was reported with a 67% (95% confidence interval [CI] 54–79) success rate (Figure 2). Regarding PETS (9 studies), the forest plot (Figure 3) showed a 76% (CI 61–89) success rate (with good result defined as ≤ 1 cm or ≤ 1.5 cm in most studies, while 2 did not report the definition of a good result). Blount staples (8 studies) was reported with a 51% (CI 28–65) success rate (Figure 4). Sensitivity analysis and forest plots for all 3 techniques including < 2 cm as acceptable for a successful result are presented in Table S9 and Figure S1 (a–c) (see Supplementary data).

### Effectiveness by other types of measurements

When considering TBP, initial LLD, ranging from 1.9–4.1 cm, and final LLD, ranging from 0.3–2.5 cm, was documented in 9 studies. The decrease in LLD varied from 0.6–2.8 cm, with 7 studies indicating a mean final LLD of < 2 cm, and 6 studies achieving < 1.5 cm final LLD (Table 2). From 283 patients treated with PETS, data on initial LLD, ranging from 1.9–3.3 cm, and final LLD, ranging from 0.3–1.7, cm was reported. In all cases, a reduction in LLD was observed. Among the 10 studies, all reported a mean final LLD of < 2 cm, with 8 studies reported a final LLD of < 1.5 cm. The decrease in LLD ranged from 1.0–2.0 cm (Table 3). Initial LLD (range 1.7–5.6), final LLD (range 0.5–2.6) and difference in LLD (range 1.1–3.0) were reported in 8 studies on Blount staples. In 6 studies mean final LLD was < 2 cm, and in 3 < 1.5 cm (Table 4).

**Table 2:** TBP with effectiveness as main outcome (successful/unsuccessful or LLD measurements initial/final and difference). LLD measurements in cm: (SD) and [range].

Study	Patients n	LLD limit ^a^	Successful		Initial LLD	Final LLD	Difference in LLD
			Yes	No			
Stevens 2022 [12]	66	≤ 1.5	38	28	NM	NM	NM
Tolk 2022 [13]	34	NM	NM	NM	2.1 (0.9)	0.8 (0.9)	1.3
Demirel 2022 [14]	11	≤ 1.5	6	5	3.9 [2.5–5.0]	2.2 [0.6–5.5]	1.7
Erdal 2022 [15]	26	≤ 1	24	2	2.4 [1.7–3.6]	0.3 [0–2.1]	2.1
De Pellegrin 2021[18]	22	≤ 1	20	2	3.7 [2–10]	NM	NM
Joeris 2017 [21]	32	= 0	21	11	NM	NM	NM
Gaumetou 2016 [22]	32	NM	NM	NM	3.0 [1.0–5.5]	1.9 [1.8–5.6]	1.1
Jochymek 2015 [23]	9	NM	7	2	2.1 [1.5–2.3]	NM	NM
Pendleton 2013 [24]	34	NM	NM	NM	1.9 [0.7–3.4]	1.1 [0–3]	0.8
Studies including 2 or more epiphysiodesis methods							
Younis 2022 [46]	17	≤ 1.5	9	8	2.6 (1.7)	1.4 (1.7)	1.3
		≤ 2	11	6			
Cheng 2021 [8]	38	NM	NM	NM	2.1 (1.0)	0.9 (0.8)	1.2
Vogt 2021 [47]	38	≤ 1	18	20	2.7[ 1.2–4.9]	NM	NM
		≤ 2	26	12			
Lee 2018 [49]	9	NM	NM	NM	3.1 (0.9)	2.5 (1.5)	0.6 (1.4)
Siedhoff 2014 [51]	4	≤ 1	2	2	2.6	1.2	1.4
		≤ 2	4	0			
Lykissas 2013 [7]	9	≤ 1.5	5	4	4.1 [2.7–5.1]	1.3 [0.4–3.3]	2.8
Total	381						
	234	examined for success of TBP					
	176	LLD measurements			1.9–4.1	0.3–2.5	0.6–2.8

a LLD limit for good outcome

NM = not mentioned.

**Table 3:** PETS with effectiveness as main outcome (successful/unsuccessful or LLD measurements initial/final and difference). LLD measurements in cm: (SD) and [range].

Study	Patients n	LLD limit ^a^	Successful		Initial LLD	Final LLD	Difference in LLD
			Yes	No			
Dodwell 2017 [25]	82	NM	79	3	2.8 [1.8–4.1]	1.7	1.0
Song 2015 [26]	48	≤ 1	44	4	1.9 [1.0–3.3]	0.3 [–1.0–1.7]	1.6
Monier 2015 [27]	16	NM	15	1	3.1 [1.3–6.0]	1.7 [0.4–3.1]	1.4
Ilharreborde 2012 [28]	45	≤ 1	21	24	ΝΜ	ΝΜ	ΝΜ
Khoury 2007 [29]	30	NM	NM	NM	2.4	1.3	1.1
Nouh 2004 [30]	9	≤ 1	5	4	3.3 [2.0–5.0]	1.4 [0.2–4.0]	1.9
Metaizeau 1999 [6]	32	≤ 1	26	6	2.5 [1.5–4.6]	0.5 [–1.1–1.3]	2.0
Studies including 2 or more epiphysiodesis methods							
Younis 2022 [46]	13	≤ 1.5	9	6	2.8 (1.5)	1.1 (1.0)	1.7
		≤ 2	12	3			
Cheng 2021 [8]	15 **^b^**	NM	NM	NM	2.2 (1.6)	1.1 (0.9)	1.1
Babu 2014 [42]	14	NM	NM	NM	3.2	1.4	1.8
Lykissas 2013 [7]	22	≤ 1.5	12	10	3.2 [2.5–4.0]	1.5 [0.7–2.4]	1.7
Campens 2010 [44]	15	≤ 1.5	11	4	NM	NM	NM
Total	343						
	284	examined for success of PETS					
	283	LLD measurements			1.9–3.3	0.3–1.7	1.0–2.0

a LLD limit for good outcome;

b Limbs.

NM = not mentioned.

**Table 4:** Staples with effectiveness as main outcome (successful/unsuccessful or LLD measurements initial/final and difference). LLD measurements in cm: (SD) and [range].

Study	Patients n	LLD limit ^a^	Successful		Initial LLD	Final LLD	Difference in LLD
			Yes	No			
Blount staples							
Gorman 2009 [3]	54	≤ 1	20	34	3.2 (1.4)	1.6 (1.3)	1.6
		≤ 2	40	14			
Skytta 2003 [31]	71	NM	NM	NM	1.8	0.5	1.3
Raab 2001 [32]	24	≤ 1	4	20	4.6	1.9	2.7
		≤ 2	14	10			
Watillon 1986 [34]	29	≤ 1	20	9	1.5–7.5	NM	NM
Mukherji 1979 [35]	47	≤ 1	33	14	2.0–7.5	NM	NM
Cabalzar 1978 [36]	78	≤ 2	31	47	5.6	2.6	3.0
May 1965 [37]	53	≤ 2	NM	NM	3.9 [1.6–6.5]	2.6	1.3
Studies including 2 or more epiphysiodesis methods							
Vogt 2021 [47]	137	≤ 1	79	58	NM	NM	1.1 [0.2–7.5]
		≤ 2	112	25			
Lee 2018 [49]	10	NM	NM	NM	1.7 (0.9)	0.2 (0.8)	1.2
Siedhoff 2014 [51]	30	≤ 1	21	9	2.3 (0.9)	0.8 (0.8)	1.5
		≤ 2	29	1			
Lykissas 2013 [7]	8	≤ 1.5	4/8	4/8	3.7 [3.4–4.0]	2.0 [0.7–4.0]	1.7
Total	541						
	407	examined for success of Blount Staples					
	328	LLD measurements			1.7–5.6	0.5–2.6	1.1–3.0
Rigid staples							
Vogt 2021 [47]							
RigidTack	45	≤ 1	23	22	NM	NM	NM
		≤ 2	36	9			
Frommer 2021 [48]							
RigidTack and							
FlexTack, TBP	58	NM	NM	NM	2.8 [2–5]	1.4 [0–4]	1.4

a LLD limit for good outcome

NM = not mentioned.

2 studies presented RigidTack and FlexTack staples [47,48], but only 1 reported success rate, with 51% (23/45) success for RigidTack (Table 4). Other outcome measurements that were reported were efficacy percentage, LLD rate correction, cost analysis, mechanical axis deviation (MAD), intra-articular morphology. and LLD ratio and were reported in 17 studies (Table S5 [a, b], see Supplementary data). Additionally, 2 studies did not define which type of epiphysiodesis was used (Table S6, see Supplementary data).

Considering the studies that compared these 3 methods, only 4 studies reported a comparison (Table S7, see Supplementary data), with 2 of them in favor of PETS over TBP, 1 in favor of staples over TBP, and 1 with no difference between TBP and PETS for LLD decrease, but with more angular deformities after TBP as a complication.

### Complications

On considering TBP usage, there were 239 complications in 453 patients (155 complications [33%] excluding as a complication poor final LLD result). PETS had 223 complications in 455 patients (162 complications [36%] excluding as a complication poor final LLD result). 516 complications were reported in 1,048 patients treated with Blount staples (381 complications [36%] excluding as a complication poor final LLD result) (Table 5).

**Table 5:** Severity grade of complications for each type of studies/techniques. Values are count (%)

Factor	Severity grade of complications ^a^				
	I	II	IIIA	IIIB	Sum
TBP (n = 453)					
Complications, n (per patient %)	50 (11)	28 (6.2)	118 (26)	43 (9.5)	239 (53)
Excluding poor LLD result			34 (5.9)		155 (33)
Not severe					78 (17)
Severe					77 (17)
PETS (n = 455)					
Complications, n (per patient %)	53 (11)	69 (15)	74 (16)	18 (4.0)	223 **^b^** (49)
Excluding poor LLD result			13 (2.9)		162 **^b^** (36)
Not severe					122 (27)
Severe					31 (6.9)
Blount staples (n = 1,048)					
Complications, n (per patient %)	107 (10)	91 (8.7)	154 (15)	143 (14)	516 **^b^** (49)
Excluding poor LLD result			19 (1.8)		381 **^b^** (36)
Not severe					198 (19)
Severe					162 (16)

a Black et al. [10].

b Not defined type of complication added.

The severe complications rate was 6.9% for PETS, 17% for TBP, and 16% for Blount staples (Table 5). Angular deformity was reported in 4% after PETS, 10% after TBP, and 17% after Blount staples (Tables 6–8). Additionally, screw removal was a complication in 51 cases (11%) with PETS treatment and reoperation due to loosening or dislocation of staples in 58 cases (5.5%) with Blount staples treatment (Tables 6–8).

**Table 6:** Number of complications for TBP according to Black [10]

Factor	I	II	IIIA	IIIB	ND
Acute complications					
Infection	1				
Effusion/edema	2				
Hematoma/hemarthrosis	1				
Knee pain	21				
Reduced knee range of motion	8				
Fracture					
Tenderness to palpation over implants	7				
Wound dehiscence/healing	2				
Skin burn/skin blistering	3				
Further surgical intervention/reoperation		6			
Peroneal nerve neuropathy					
Long-term complications					
Failure of growth plate arrest			7		
Failure to achieve adequate reduction in					
LLD (according to definition of poor result)			84		
Overcorrection			2		
Angular deformity, varus				11	
Angular deformity, valgus				20	
Angular deformity (not defining what type)				12	
Genu recurvatum					
Asymmetrical closure of the growth plate					
with progressive malalignment					
Exostosis					
Rebound overgrowth					
TBP, screw bending without problem removal	5				
TBP, screw migrated during correction		3			
TBP, reinsertion of screw					
(readmission for surgery)		10			
TBP, breakage screw		3			
TBP, 1 of the 3 above		6			
Neurapraxia					
Additional surgeries needed for LLD treatment			25		
Complications undefined (can’t be categorized)					
Total (n = 239) **^a^**	50	28	118	43	0

ND: not defined complications.

a 155 complications (33%) excluding as a complication poor final LLD result.

**Table 7:** Number of complications for PETS according to Black [10]

Factor	I	II	IIIA	IIIB	ND
Acute complications					
Infection					
Effusion/edema	13				
Hematoma/hemarthrosis	2				
Knee pain	25				
Reduced knee range of motion					
Fracture					
Wound dehiscence/healing	3				
Skin burn/skin blistering					
Further surgical intervention/reoperation		1			
Peroneal nerve neuropathy					
Long-term complications					
Failure of growth plate arrest			2		
Failure to achieve adequate reduction in					
LLD (according to definition of poor result)			61		
Overcorrection			2		
Angular deformity, varus					
Angular deformit, valgus				10	
Angular deformity (not defining what type)/					
axial deviation				7	
Genu recurvatum				1	
Asymmetrical closure of the growth plate					
with progressive malalignment					
Exostosis					
Rebound overgrowth					
Broken screw		4			
Symptomatic screw removal		51			
Screw issues during removal	10				
Screw dislodged from epiphysis		3			
Revision surgery for screw reposition		10			
Neurapraxia					
Additional surgeries needed for LLD treatment			9		
Complications undefined (can’t be categorized)					9
Total (n = 223) **^a^**	53	69	74	18	9

ND: not defined complications,

a 162 complications (36%) excluding as a complication poor final LLD result.

**Table 8:** Number of complications for staples according to Black [10]

Factor	I	II	IIIA	IIIB	ND
Acute complications					
Infection	2	9			
Effusion/edema	18				
Hematoma/hemarthrosis					
Knee pain	4				
Reduced knee range of motion	59				
Fracture					
Irritation from prominent staples	9				
Wound dehiscence/healing	8				
Skin burn/skin blistering					
Further surgical intervention/reoperation					
Peroneal nerve neuropathy/neurovascular	1				
Long-term complications					
Failure of growth plate arrest			9		
Failure to achieve adequate reduction in					
LLD (according to definition of poor result)			135		
Overcorrection			6		
Angular deformity, varus				45	
Angular deformity, valgus				33	
Angular deformity (not defining what type)/					
axial deviation				20	
Genu recurvatum				42	
Asymmetrical closure of the growth plate					
with progressive malalignment					
Exostosis					
Rebound overgrowth			2		
Backed staples but no surgery	5				
Reoperation due to loosening or					
dislocation of staples		58			
Retained staples that could not be removed	1				
Broken staples		4			
Implant-associated (breakage, loosening,					
migration)		20			
Neurapraxia				3	
Additional surgeries needed for LLD treatment			2		
Complications undefined (can’t be categorized)					21
Total (n = 516) **^a^**	107	91	154	143	21

ND: not defined complications.

a 381 complications (36%) excluding as a complication poor final LLD result.

## Discussion

This is the first systematic review and proportional meta-analysis to summarize current knowledge on LLD treatment using epiphysiodesis with an implant. We aimed to evaluate the effectiveness of 3 different epiphysiodesis techniques with implant usage for the treatment of LLD in the pediatric population and to address the reported complications of staples, TBP, and PETS. We showed a 76% success rate for PETS, but only 67% and 51% for TBP and Blount staples, respectively. Additionally, the severe complications rate was 7% for PETS, 17% for TBP, and 16% for Blount staples, with Blount and TBP use leading to a greater number of angular deformities. It seems that all techniques may have the potential of being successful in growth inhibition with mean success rates ranging from 51% to 76%. Differences were found in both crude success and complication rates between the studies, but the lack of randomized studies does not allow for firm conclusions regarding which technique is superior.

The use of Blount staples has been lessened through the years because, as also presented in the current systematic review, the technique has been connected to complications such as propensity for breakage, loosening, migration, and asymmetric growth [52]. Looking at TBP use, one possible drawback of using guided growth for epiphysiodesis is that it might impede peripheral physeal growth while not affecting central growth, possibly resulting in an irregular, mountain-shaped physis [1,20].

In terms of the complications, we did not identify any reduced knee range motion in the PETS group. This might be due to the heterogeneity on reporting complications, and due to the fact that in none of those studies were complications the study’s main outcome. On the contrary, the large amount of cases with reduced knee range of motion after staples (see Table 8) might indicate a more invasive procedure than the other 2 techniques where fewer implants are used. Another reason could be that the size of the sample was almost 2 times larger for staples than for PETS.

Comparing the results of the current study with those in our previous published systematic review [53] on the use of percutaneous epiphysiodesis (PE) and the Phemister technique, 2 surgical techniques with permanent effect, PETS seems to have the best overall success rate, as PE had a 74% success rate and the Phemister technique a 69% success rate. However, PE was found to have less severe complications (5.1%), followed by PETS. The need for future randomized controlled trials that compare PETS with PE is highlighted by the results of our studies, as well as the importance of improved reporting regarding preoperative planning, particularly considering that the timing of surgery significantly impacts outcomes in reducing length differences.

### Strengths and limitations

***Strengths.*** There were no language restrictions, thus reducing reporting bias. Grading the severity of the complications by the classification of Black et al. [10], a proportional meta-analysis was suitable to analyze the effectiveness and success rate, as it is a tool for synthesizing proportion-based data, offering a more precise, reliable, and generalizable understanding of the research question.

***Limitations.*** The variations in methodology among the studies, particularly in terms of study design, timing of interventions, follow-up procedures, and reporting of results, posed difficulties in generalizing the results. However, the identification of heterogeneity in all the above-mentioned factors is of great importance, because it highlights the need for more detailed and standardized way of reporting in studies on the topic, and especially considering the reporting of complications. Some questions that arise and should be taken into consideration for future studies are the following: studies seem to have limited information regarding the patients undergoing second surgery after implant removal following correction and skeletal maturity; only a few studies reported skeletal age at time of insertion and did not provide enough information regarding timing of the procedure as well as effectiveness of physeal inhibition.

The sample sizes were small and the nature of the evidence from most of the included studies was retrospective, with a lower level of quality for the non-comparing studies. As a result, the results of the pooled analysis cannot be generalized, especially the results for complication rates. Bias could also emerge from variations in techniques (such as screw positioning in TBP or PETS), the absence of assessments at full maturity, and disparities in the methods used for the initial evaluation of LLD.

### Conclusion

PETS appears to be the most effective form of epiphysiodesis surgery involving an implant, exhibiting a superior success rate and fewer severe complications like angular deformities compared with TBP or Blount staples.

In perspective, future studies should focus on better reporting of methods and outcomes, as well as on conducting randomized clinical trials on the topic.

### Supplementary data

Tables S1–S9, Figures S1 (a–c) and search criteria are available as Supplementary data on the article page, doi: 10.2340/17453674.2024.41104

## Supplementary Material



## Notes

MT: study design, literature search, extraction of data, risk of bias assessment, statistical analysis, manuscript preparation. MHH: literature search, extraction of data. JFJ: literature search, risk of bias assessment. SK: manuscript preparation, supervision. OR: study design, manuscript preparation, supervision.

Handling co-editors: Ilkka Helenius and Robin Christensen

*Acta* thanks Klaus Dieter Parsch, Michael Sussman, and Li Zhao for help with peer review of this manuscript.

## References

[1] Ruzbarsky J J, Goodbody C, Dodwell E. Closing the growth plate: a review of indications and surgical options. Curr Opin Pediatr 2017; 29(1): 80-6. doi: 10.1097/MOP.0000000000000438. 27845969

[2] Blount W P, Clarke G R. Control of bone growth by epiphyseal stapling; a preliminary report. J Bone Joint Surg Am 1949; 31(3): 464-78. 18153890

[3] Gorman T M, Vanderwerff R, Pond M, MacWilliams B, Santora S D. Mechanical axis following staple epiphysiodesis for limb-length inequality. J Bone Joint Surg Am 2009; 91(10): 2430-9. doi: 10.2106/JBJS.H.00896. 19797579

[4] Stevens P. Guided growth: 1933 to the present. Strateg Trauma Limb Reconstr 2006; 1: 29-35. doi: 10.1007/s11751-006-0003-3.

[5] Gottliebsen M, Shiguetomi-Medina J M, Rahbek O, Møller-Madsen B. Guided growth: mechanism and reversibility of modulation. J Child Orthop 2016;10(6):471-477. doi: 10.1007/s11832-016-0778-9. 27826908 PMC5145828

[6] Metaizeau J P, Wong-Chung J, Bertrand H, Pasquier P. Percutaneous epiphysiodesis using transphyseal screws (PETS). J Pediatr Orthop 1998; 18(3): 363-9. 9600565

[7] Lykissas M G, Jain V V, Manickam V, Nathan S, Eismann E A, McCarthy J J. Guided growth for the treatment of limb length discrepancy: a comparative study of the three most commonly used surgical techniques. J Pediatr Orthop B 2013; 22(4): 311-17. doi: 10.1097/BPB.0b013e32836132f0. 23588389

[8] Cheng Y-H, Lee W-C, Tsai Y-F, Kao H-K, Yang W-E, Chang C-H. Tension band plates have greater risks of complications in temporary epiphysiodesis. J Child Orthop 2021; 15: 106-13. doi: 10.1302/1863-2548.15.200180. 34040656 PMC8138785

[9] Page M J, McKenzie J E, Bossuyt P M, Boutron I, Hoffmann T C, Mulrow C D et al. The PRISMA 2020 statement: an updated guideline for reporting systematic reviews. Syst Rev 2021; 10: 89. doi: 10.1136/bmj.n71. 33781348 PMC8008539

[10] Black S R, Kwon M S, Cherkashin A M, Samchukov M L, Birch J G, Jo C H. Lengthening in congenital femoral deficiency: a comparison of circular external fixation and a motorized intramedullary nail. J Bone Joint Surg Am 2015; 97(17): 1432-40. doi: 10.2106/JBJS.N.00932 26333739 PMC7535106

[11] Slim K, Nini E, Forestier D, Kwiatkowski F, Panis Y, Chipponi J. Methodological index for non-randomized studies (minors): development and validation of a new instrument. ANZ J Surg 2003; 73(9): 712-16. doi:10.1046/j.1445-2197.2003.02748.x. 12956787

[12] Stevens P, Desperes M, McClure P K, Presson A, Herrick J. Growth deceleration for limb length discrepancy: tension band plates followed to maturity. Strategies Trauma Limb Reconstr 2022; 17(1): 26-31. doi: 10.5005/jp-journals-10080-1548. 35734037 PMC9166257

[13] Tolk J J, Merchant R, Calder P R, Hashemi-Nejad A, Eastwood D M. Tension-band plating for leg-length discrepancy correction. Strategies Trauma Limb Reconstr 2022; 17(1): 19-25. doi: 10.5005/jp-journals-10080-1547. 35734032 PMC9166256

[14] Demirel M, Sağlam Y, Yıldırım A M, Bilgili F, Şeker A, Şen C. Temporary epiphysiodesis using the eight-plate in the management of children with leg length discrepancy: a retrospective case series. Indian J Orthop 2022; 56(5): 874-882. doi: 10.1007/s43465-021-00599-9. 35547335 PMC9043087

[15] Erdal O A, Gorgun B, Razi O, Sarikaya I A, Inan M. Effects of tension band plating on coronal plane alignment of lower extremities in children treated for idiopathic limb length discrepancy. J Child Orthop 2022; 16(6): 505-11. doi: 10.1177/18632521221135192. 36483641 PMC9723863

[16] Petrova D A, Kenis V M. Assessment of comparative parameters of leg length discrepancy in children using temporary epiphysiodesis with 8-plates. Ped Traum Orthop Rec Surg 2022; 10(2): 151-60. doi: 10.17816/PTORS104405.

[17] Ozdemir E, Cetik R M, Ayvaz M, Yilmaz G. The efficacy of two-hole tension band plates in the treatment of lower extremity limb length discrepancy. J Pediatr Orthop B 2022; 31(1): e31-e36. doi: 10.1097/BPB.0000000000000861. 33720078

[18] De Pellegrin M, Brogioni L, Laskow G, Barera G, Pajno R, Osimani S, et al. Guided growth in leg length discrepancy in Beckwith-Wiedemann Syndrome: a consecutive case series. Children (Basel) 2021; 8(12): 1152. doi: 10.3390/children8121152. 34943348 PMC8700625

[19] Masquijo J, Allende V, Artigas C, Hernández Bueno J C, Morovic M, Sepúlveda M. Partial hardware removal in guided growth surgery: a convenient strategy? Rev Esp Cir Ortop Traumatol (Engl Ed) 2021; 65(3): 195-200. doi: 10.1016/j.recot.2020.09.003. 33419673

[20] Sinha R, Weigl D, Mercado E, Becker T, Kedem P, Bar-On E. Eight-plate epiphysiodesis: are we creating an intra-articular deformity? Bone Joint J 2018; 100-B(8): 1112-16. doi: 10.1302/0301-620X.100B8.BJJ-2017-1206.R3. 30062943

[21] Joeris A, Ramseier L, Langendörfer M, von Knobloch M, Patwardhan S, Dwyer J, et al. Paediatric lower limb deformity correction with the Eight Plate: adverse events and correction outcomes of 126 patients from an international multicentre study. J Pediatr Orthop B 2017; 26(5): 441-8. doi: 10.1097/BPB.0000000000000397. 27832012

[22] Gaumétou E, Mallet C, Souchet P, Mazda K, Ilharreborde B. Poor efficiency of eight-plates in the treatment of lower limb discrepancy. J Pediatr Orthop 2016; 36(7): 715-19. doi: 10.1097/BPO.0000000000000518. 25988679

[23] Jochymek J, Peterková T. [Eight-plate guided growth treatment for angular deformities and length discrepancies of the lower limbs in children:. our first experience]. Acta Chir Orthop Traumatol Cech 2015; 82(6): 424-9. 26787183

[24] Pendleton A M, Stevens P M, Hung M. Guided growth for the treatment of moderate leg-length discrepancy. Orthopedics 2013; 36(5):e575-80. doi: 10.3928/01477447-20130426-18. 23672908

[25] Dodwell E R, Garner M R, Bixby E, Luderowski E M, Green D W, Blanco J S, et al. Percutaneous epiphysiodesis using transphyseal screws: a case series demonstrating high efficacy. HSS J 2017; 13(3): 255-62. doi: 10.1007/s11420-017-9549-5. 28983218 PMC5617815

[26] Song M H, Choi E S, Park M S, Yoo W J, Chung C Y, Choi I H, et al. Percutaneous epiphysiodesis using transphyseal screws in the management of leg length discrepancy: optimal operation timing and techniques to avoid complications. J Pediatr Orthop 2015; 35(1): 89-93. doi: 10.1097/BPO.0000000000000214. 24978321

[27] Monier B C, Aronsson D D, Sun M. Percutaneous epiphysiodesis using transphyseal screws for limb-length discrepancies: high variability among growth predictor models. J Child Orthop 2015; 9(5): 403-10. doi: 10.1007/s11832-015-0687-3. 26423270 PMC4619365

[28] Ilharreborde B, Gaumetou E, Souchet P, Fitoussi F, Presedo A, Penneçot G F, et al. Efficacy and late complications of percutaneous epiphysiodesis with transphyseal screws. J Bone Joint Surg Br 2012; 94(2): 270-5. doi: 10.1302/0301-620X.94B2.27470. 22323699

[29] Khoury J G, Tavares J O, McConnell S, Zeiders G, Sanders J O. Results of screw epiphysiodesis for the treatment of limb length discrepancy and angular deformity. J Pediatr Orthop 2007; 27(6): 623-8. doi: 10.1097/BPO.0b013e318093f4f4. 17717460

[30] Nouth F, Kuo L A. Percutaneous epiphysiodesis using transphyseal screws (PETS): prospective case study and review. J Pediatr Orthop 2004; 24(6): 721-5. 15502577

[31] Skyttä E, Savolainen A, Kautiainen H, Lehtinen J, Belt E A. Treatment of leg length discrepancy with temporary epiphyseal stapling in children with juvenile idiopathic arthritis during 1957–99. J Pediatr Orthop 2003; 23(3): 378-80. 12724604

[32] Raab P, Wild A, Seller K, Krauspe R. Correction of length discrepancies and angular deformities of the leg by Blount’s epiphyseal stapling. Eur J Pediatr 2001; 160(11): 668-74. doi: 10.1007/s004310100834. 11760024

[33] Sengupta A, Gupta P. Epiphyseal stapling for leg equalization in developing countries. Int Orthop 1993; 17(1): 37-42. doi: 10.1007/BF00195222. 8449622

[34] Watillon M, Hoet F. [Epiphyseal stapling in the treatment of leg length inequality]. Acta Orthop Belg 1986; 52(2): 209-16. 3739679

[35] Mukherji D, Das A K. Epiphyseal stapling for the correction of lower limb inequality following poliomyelitis. Indian J Orthop 1979; 13(1): 17-22.

[36] Cabalzar A. [Experiences with temporary epiphyseal stapling by Blount]. Z Orthop Ihre Grenzgeb 1978; 116(3): 355-62. 685391

[37] May V R Jr, Clements E L. Epiphyseal stapling: with special reference to complications. South Med J 1965; 58(10): 1203-7. doi: 10.1097/00007611-196510000-00002. 4284697

[38] Cohen L L, Shore B J, Miller P E, Troy M J, Mahan S T, Kasser J R, et al. Epiphysiodesis for leg length discrepancy: a cost analysis of drill versus screw technique. J Surg Orthop Adv 2021; 30(3) :181-4. 34591010

[39] Borbas P, Agten C A, Rosskopf A B, Hingsammer A, Eid K, Ramseier L E. Guided growth with tension band plate or definitive epiphysiodesis for treatment of limb length discrepancy? J Orthop Surg Res 2019; 14(1): 99. doi: 10.1186/s13018-019-1139-4. 30971266 PMC6458784

[40] Troy M, Shore B, Miller P, Mahan S, Hedequist D, Heyworth B, et al. A comparison of screw versus drill and curettage epiphysiodesis to correct leg-length discrepancy. J Child Orthop 2018; 12(5): 509-14. doi: 10.1302/1863-2548.12.180030. 30294377 PMC6169556

[41] Bayhan I A, Karatas A F, Rogers K J, Bowen J R, Thacker M M. Comparing percutaneous physeal epiphysiodesis and eight-plate epiphysiodesis for the treatment of limb length discrepancy. J Pediatr Orthop 2017; 37(5): 323-7. doi: 10.1097/BPO.0000000000000647. 26368859

[42] Babu L V, Evans O, Sankar A, Davies A G, Jones S, Fernandes J A. Epiphysiodesis for limb length discrepancy: a comparison of two methods. Strategies Trauma Limb Reconstr 2014; 9(1): 1-3. doi: 10.1007/s11751-013-0180-9. 24271553 PMC3951623

[43] Stewart D, Cheema A, Szalay E A. Dual 8-plate technique is not as effective as ablation for epiphysiodesis about the knee. J Pediatr Orthop 2013; 33(8): 843-6. doi: 10.1097/BPO.0b013e3182a11d23. 23872800

[44] Campens C, Mousny M, Docquier P L. Comparison of three surgical epiphysiodesis techniques for the treatment of lower limb length discrepancy. Acta Orthop Belg 2010; 76(2): 226-32. 20503949

[45] Frediani P, Nocivelli P, Capilupi P. [Role of epiphysiodesis in the treatment of leg length inequality]. Chir Organi Mov 1987; 72: 221–6. 3436186

[46] Younis M H, Hanstein R, Javed K, Fornari E D, Gomez J A, Sharkey M S, et al. Percutaneous epiphysiodesis using transphyseal screws (PETS) versus tension-band plating (TBP): comparative study of outcomes for correcting limb length discrepancy. Eur J Orthop Surg Traum 2023; 33(5): 1523-31. doi: 10.1007/s00590-022-03304-0. 35723838

[47] Vogt B, Roedl R, Gosheger G, Frommer A, Laufer A, Kleine-Koenig M T, et al. Growth arrest: leg length correction through temporary epiphysiodesis with a novel rigid staple (RigidTack). Bone Joint J 2021; 103-B(8): 1428-37. doi: 10.1302/0301-620X.103B8.BJJ-2020-1035.R4. 34334047 PMC9948429

[48] Frommer A, Niemann M, Gosheger G, Eveslage M, Toporowski G, Laufer A, et al. Temporary proximal tibial epiphysiodesis for correction of leg length discrepancy in children: should proximal fibular epiphysiodesis be performed concomitantly? J Clin Med 2021; 10(6): 1245. doi: 10.3390/jcm10061245. 33802874 PMC8002647

[49] Lee W C, Kao H K, Yang W E, Chang C H. Tension band plating is less effective in achieving equalization of leg length. J Child Orthop 2018; 12(6): 629-34. doi: 10.1302/1863-2548.12.170219. 30607211 PMC6293331

[50] Corradin M, Schiavon R, Borgo A, Deslandes J, Cersosimo A, Canavese F. The effects of uninvolved side epiphysiodesis for limb length equalization in children with unilateral cerebral palsy: clinical evaluation with the Edinburgh visual gait score. Eur J Orthop Surg Traumatol 2018; 28: 977–84. doi: 10.1007/s00590-017-2097-3. 29214458

[51] Siedhoff M, Ridderbusch K, Breyer S, Stücker R, Rupprecht M. Temporary epiphysiodesis for limb-length discrepancy: 8- to 15-year follow-up of 34 children. Acta Orthop 2014; 85(6): 626-32. doi: 10.3109/17453674.2014.960646. 25191935 PMC4259036

[52] Bylander B, Hansson L I, Selvik G. Pattern of growth retardation after Blount stapling: a roentgen stereophotogrammetric analysis. J Pediatr Orthop 1983; 3(1): 63-72. doi: 10.1097/01241398-198302000-00011. 6841605

[53] Tirta M, Hjorth M H, Jepsen J F, Rahbek O, Kold S. Are percutaneous epiphysiodesis and Phemister technique effective in the treatment of leg-length discrepancy? A systematic review. J Pediatr Orthop B 2024. doi: 10.1097/BPB.0000000000001160. PMC11444351 38324644

