# Supplementary material for: Staples, tension-band plates, and percutaneous epiphysiodesis screws used for leg-length discrepancy treatment: a systematic review and proportional meta-analysis
Source: Acta Orthop. 2024 Jul 18;95:415–24. doi: 10.2340/17453674.2024.41104 (PMC11257069; doi:10.2340/17453674.2024.41104)

Supplementary Material

Table S1. Search strategy for the five electronic databases

(a) PubMed

Search	Query
#13	Search: (((("Leg Length Inequality"[Mesh]) OR (leg length discrepanc*[Text Word] OR leg length inequalit*[Text Word] OR leg length correcti*[Text Word])) OR (limb length discrepanc*[Text Word] OR limb length inequalit*[Text Word] OR limb length correcti*[Text Word])) OR (lower limb discrepanc*[Text Word] OR lower limb inequalit*[Text Word] OR lower limb correcti*[Text Word])) AND (((((("Epiphyses/surgery"[Mesh]) OR (epiphysiodes*[Text Word])) OR (physiodesis[Text Word])) OR (tension band plating[Text Word])) OR (eight plate*[Text Word])) OR (guided growth[Text Word])) Sort by: Publication Date
#12	Search: ((((("Epiphyses/surgery"[Mesh]) OR (epiphysiodes*[Text Word])) OR (physiodesis[Text Word])) OR (tension band plating[Text Word])) OR (eight plate*[Text Word])) OR (guided growth[Text Word]) Sort by: Publication Date
#11	Search: guided growth[Text Word] Sort by: Publication Date
#10	Search: eight plate*[Text Word] Sort by: Publication Date
#9	Search: tension band plating[Text Word] Sort by: Publication Date
#8	Search: physiodesis[Text Word] Sort by: Publication Date
#7	Search: epiphysiodes*[Text Word] Sort by: Publication Date
#6	Search: "Epiphyses/surgery"[Mesh] Sort by: Publication Date
#5	Search: ((("Leg Length Inequality"[Mesh]) OR (leg length discrepanc*[Text Word] OR leg length inequalit*[Text Word] OR leg length correcti*[Text Word])) OR (limb length discrepanc*[Text Word] OR limb length inequalit*[Text Word] OR limb length correcti*[Text Word])) OR (lower limb discrepanc*[Text Word] OR lower limb inequalit*[Text Word] OR lower limb correcti*[Text Word]) Sort by: Publication Date
#4	Search: lower limb discrepanc*[Text Word] OR lower limb inequalit*[Text Word] OR lower limb correcti*[Text Word] Sort by: Publication Date
#3	Search: limb length discrepanc*[Text Word] OR limb length inequalit*[Text Word] OR limb length correcti*[Text Word] Sort by: Publication Date
#2	Search: leg length discrepanc*[Text Word] OR leg length inequalit*[Text Word] OR leg length correcti*[Text Word] Sort by: Publication Date
#1	Search: "Leg Length Inequality"[Mesh] Sort by: Publication Date

(b) Embase

No.	Query
#11	#3 AND #10
#10	#4 OR #5 OR #6 OR #7 OR #8 OR #9
#9	'guided growth':ti,ab,kw
#8	'eight plate*':ti,ab,kw
#7	'tension band plating':ti,ab,kw
#6	physiodesis:ti,ab,kw
#5	epiphysiodes*:ti,ab,kw
#4	'epiphysiodesis'/de
#3	#1 OR #2
#2	((leg OR limb) NEAR/2 (discrepanc* OR inequalit* OR correcti*)):ti,ab,kw
#1	'leg length inequality'/de

(c) Cochrane

ID	Search
#1	MeSH descriptor: [Leg Length Inequality] explode all trees
#2	((leg OR limb) NEAR/2 (discrepanc* OR inequalit* OR correcti*)):ti,ab,kw
#3	#1 or #2
#4	MeSH descriptor: [Epiphyses] explode all trees and with qualifier(s): [surgery - SU]
#5	(epiphysiodes* or physiodesis):ti,ab,kw
#6	(tension band plating):ti,ab,kw
#7	(eight NEXT plate*):ti,ab,kw
#8	(guided growth):ti,ab,kw
#9	{OR #4-#8}
#10	#3 and #9

(d) Web of Science

#	Search
#7	#1 AND #6
#6	#2 OR #3 OR #4 OR #5
#5	guided growth (Topic)
#4	eight plate* (Topic)
#3	tension band plating (Topic)
#2	epiphysiodesis OR physiodesis (Topic)
#1	(leg OR limb) NEAR/2 (discrepanc* OR inequalit* OR correcti*) (Topic)

(e) Scopus

#	Search
10	((TITLE-ABS-KEY ("leg length discrepanc*" OR "leg length inequalit*" OR "leg length correcti*")) OR (TITLE-ABS-KEY ("limb length discrepanc*" OR "limb length inequalit*" OR "limb length correcti*")) OR (TITLE-ABS-KEY ("lower limb discrepanc*" OR "lower limb inequalit*" OR "lower limb correcti*"))) AND ((TITLE-ABS-KEY (epiphysiodes* OR physiodesis)) OR (TITLE-ABS-KEY ("tension band plating")) OR (TITLE-ABS-KEY ("eight plate*")) OR (TITLE-ABS-KEY ("guided growth")))
9	(TITLE-ABS-KEY (epiphysiodes* OR physiodesis)) OR (TITLE-ABS-KEY ("tension band plating")) OR (TITLE-ABS-KEY ("eight plate*")) OR (TITLE-ABS-KEY ("guided growth"))
8	TITLE-ABS-KEY ("guided growth")
7	TITLE-ABS-KEY ("eight plate*")
6	TITLE-ABS-KEY ("tension band plating")
5	TITLE-ABS-KEY (epiphysiodes* OR physiodesis)
4	(TITLE-ABS-KEY ("leg length discrepanc*" OR "leg length inequalit*" OR "leg length correcti*")) OR (TITLE-ABS-KEY ("limb length discrepanc*" OR "limb length inequalit*" OR "limb length correcti*")) OR (TITLE-ABS-KEY ("lower limb discrepanc*" OR "lower limb inequalit*" OR "lower limb correcti*"))
3	TITLE-ABS-KEY ("lower limb discrepanc*" OR "lower limb inequalit*" OR "lower limb correcti*")
2	TITLE-ABS-KEY ("limb length discrepanc*" OR "limb length inequalit*" OR "limb length correcti*")
1	TITLE-ABS-KEY ("leg length discrepanc*" OR "leg length inequalit*" OR "leg length correcti*")

Table S2. Studies excluded at full text review.

Reference		Reason
**1.**	Boyle J, Makarov MR, Podeszwa DA, Rodgers JA, Jo CH, Birch JG; Is Proximal Fibula Epiphysiodesis Necessary When Performing a Proximal Tibial Epiphysiodesis. J Article United States J Pediatr Orthop. 2020 Nov/Dec;40(10):e984-e989.	Fibular overgrowth
**2.**	Bredemeier H, Dahmen G; Treatment of inequality in the length of the legs. Hippokrates 1973;44(4):464-466.	Review
**3.**	Fillingham YA, Kogan M; Epiphysiodesis for Limb Length Discrepancy and Angular Deformity. Case Competencies in Orthopaedic Surgery. 2016;195-207.	Review
**4.**	Johnston CE 2nd, Bueche MJ, Williamson B, Birch JG. Epiphysiodesis for management of lower limb deformities. Instr Course Lect. 1992;41:437-44.	Review
**5.**	Kingma, MJ; Epiphysiodesis. Maandschr Kindergeneeskd 1966;34(1): 34-9.	Review
**6.**	Lampe HI, Swierstra BA, Diepstraten AF. Timing of physiodesis in limb length inequality. The Straight Line Graph applied in 30 patients. Acta Orthop Scand. 1992 Dec;63(6):672-4.	Not exact epiphysiodesis technique defined
**7.**	McCarthy JJ, Burke T, McCarthy MC. Need for concomitant proximal fibular epiphysiodesis when performing a proximal tibial epiphysiodesis. J Pediatr Orthop. 2003 Jan-Feb;23(1):52-4.	Fibular overgrowth
**8.**	Sepúlveda OM, Ferrada P, Miranda-Chacón Z, Saban GE, Pérez G J. Evolución de la Cirugía de Epifisiodesis [Evolution of Epiphysiodesis Surgery]. Andes Pediatr. 2021 Oct;92(5):733-738.	Not exact epiphysiodesis technique defined
**9.**	Stephens DC, Herrick W, MacEwen GD. Epiphysiodesis for limb length inequality: results and indications. Clin Orthop Relat Res. 1978 Oct;(136):41-8.	Not exact epiphysiodesis technique defined
**10.**	Wehbe J, Maalouf G, Morin C; Action to be taken when confronted with leg length inequality: Synthesis of a series of 240 cases. Revue Medicale Libanaise 2003;15(3):138-141.	Opinion/Experience
**11.**	Blount WP. A mature look at epiphyseal stapling. Clin Orthop Relat Res. 1971; 77:158-63.	Case Report
**12.**	Blount WP, Clarke GR. The classic. Control of bone growth by epiphyseal stapling. A preliminary report. Journal of Bone and Joint Surgery, July 1949. Clin Orthop Relat Res. 1971; 77:4-17	Experience/Technique
**13.**	Chen SC. Long-term results of stapling of the knee. Proc R Soc Med. 1970 Aug;63(8):755-6	Not enough information
**14.**	Lauge-Pedersen H, Hägglund G. Eight plate should not be used for treating leg length discrepancy. J Child Orthop. 2013 Oct;7(4):285-8.	Not enough information
**15.**	De Arrascaeta, D, Perez, M. Surgical Treatment of Lower Limb Length Discrepancy and Angular Deformity by Epiphysiodesis with Transphyseal Screws (Metaizeau Technique) Anal Facult Medicina 2017; 4(1),137-15	Not enough information

Table S3. Characteristics of included studies					
**Study Characteristics**	**Inclusion/ Exclusion criteria**	**Assessment pre-operatively**	**Surgery details**	**Patients Characteristics**	**Follow-up duration**
**Tension-band plates (TBP)**					
**Stevens 2022 [12]**<br>Country: USA<br>Type of study: Retrospective observational study (Case-series)<br>Duration: 2005-2017<br>Epiphysiodesis: TBP	**Inclusion criteria:** Patients with (1) predicted discrepancy of 2–9 cm at maturity; (2) minimum of 1 year of predicted growth remaining at the time of epiphysiodesis; (3) minimum 18-month follow-up and (4) minimum Risser stage I (R1) at the last radiologic study, reflecting impending skeletal maturity.<br>**Exclusion criteria:** Patients with previous growth modulation history for coronal deformity treatment or insufficient radiologic follow-up or both	Assessment: Teleroentgenogram with block under the foot<br>Skeletal Maturity: Girls at age 14 and boys at age 16, hand X-ray<br>Prediction of LLD at maturity/ Timing: White-Menelaus method, LLD 2 cm or at least a year earlier than skeletal maturity	Post-operative: Full weightbearing, physical therapy<br><br>Removal: At maturity or upon achieving the desired correction	Total: 66 patients<br>Sex: M 32/ F 34<br>Age: 3-16.6 yrs<br>Etiology: Idiopathic LLD<br>Bone: Femur 35, Tibia 5, Femur/Tibia 26<br>Side: NM	Maturity
**Tolk 2022 [13]**<br>Country: UK<br>Type of study: Retrospective observational study (Case-series)<br>Duration: 2012-2020<br>Epiphysiodesis: TBP	**Inclusion criteria:** Patients with dual TBP for correction of LLD, on either the distal femur or proximal tibia or both.<br>**Exclusion criteria:** If appropriate long leg films were not available before and after LLD or if they had undergone any other leg-length correction procedure during the guided growth period. Those that had had a previous or concomitant injury or intervention to the contralateral proximal tibia or distal femoral physes that would affect longitudinal growth.	Assessment: Standardized long-leg radiographs<br>Skeletal Maturity: Bilateral closure of both the distal femoral and proximal tibial physes on radiographic evaluation.<br>Prediction of LLD at maturity/Timing: NM	Post-operative: Full weightbearing<br><br>Removal: NM	Total: 34 patients<br>Sex: M 9/ F 25<br>Age: 12.1 yrs (±1.7)<br>Etiology: DDH, Perthes<br>Bone: Femur 24, Tibia 6, Femur/Tibia 4<br>Side: R 15/ L 19	Maturity

**Demirel 2022 [14]**<br>Country: Turkey<br>Type of study:<br>Case series<br>Duration: 2010-2018<br>Epiphysiodesis: TBP	**Inclusion criteria:**<br>(1)A diagnosis of LLD of up to 5 cm, (2) Treated by temporary epiphysiodesis using TBP, (3) Adequate clinical and radiographic follow-up until skeletal maturity, (4) A minimum of 24 months follow-up after the index surgery, (5) Being willing to participate in the study.<br>**Exclusion criteria:** (1) Lost to follow-up, (2) Incomplete medical records and radiographic images, (3) A history of previous lower limb correction surgery, (4) Being unwilling to participate in the study<br>.	Assessment:<br>Standing radiographs in both coronal and sagittal planes<br>Skeletal Maturity: Girls at age 14 and boys at age 16<br>Prediction of LLD at maturity/Timing: Anderson and Green growth remaining charts and the Paley multiplier method	Post-operative:<br>Full weightbearing<br><br>Removal: At skeletal maturity or when the leg length was balanced	Total: 11 patients<br>Sex: 7 M/ 4 F<br>Age: 9 yrs (6-11)<br>Etiology: Idiopathic LLD<br>Bone: Femur 3, Tibia 5, Femur/Tibia 3<br>Side: R 8/ L 3	Maturity, 62 mos (39–106)
**Erdal 2022 [15]**<br>Country: Turkey<br>Type of study:<br>Retrospective observational study (Case-series)<br>Duration: 2013-2019<br>Epiphysiodesis: TBP	**Inclusion criteria:** Cases idiopathic LLD<br>**Exclusion criteria:** Non-idiopathic cases, patients lost during follow-up, revision of cases from another institution, cases with previous lower extremity deformity, and those that required concomitant osteotomy or TBP at a level other than the knee were excluded from the study**.**	Assessment:<br>Full-length leg radiographs<br>Skeletal Maturity: NM<br>Prediction of LLD at maturity/Timing: NM	Post-operative: NM<br><br>Removal: Plates were removed when LLD was eliminated.	Total: 26 patients<br>Sex: NM<br>Age: 11 yrs (6-14)<br>Etiology: Idiopathic LLD<br>Bone: Femur 12, Tibia 3, Femur/Tibia 11<br>Side: NM	Maturity, 61.5 mos (25–94)
**Petrova 2022 [16]**<br>Country: Russia<br>Type of study:<br>Retrospective observational study (Case-series)<br>Duration: 2015-2020<br>Epiphysiodesis: TBP	**Inclusion criteria:** Patients that undergone surgery TBP for LLD<br>**Exclusion criteria:** NM	Assessment:<br>Standardized long-leg radiographs<br>Skeletal Maturity: NM<br>Prediction of LLD at maturity/Timing: NM	Two 8-plates both sides (medial & lateral)<br><br>Post-operative: NM<br><br>Removal: NM	Total: 94 patients<br>Sex: 56 M/38 F<br>Age: NM<br>Etiology: Congenital<br>Bone: Femur 64, Tibia 38<br>Side: NM	NM

**Ozdemir 2021[17]**<br>Country: Turkey<br>Type of study: Retrospective observational study (Case-series)<br>Duration: 1/2012-6/2018<br>Epiphysiodesis: TBP	**Inclusion criteria:** Patients who had epiphysiodesis utilizing dual TBP and had at least 18 months follow-up period with full-length lower extremity weight-bearing radiographs.<br>**Exclusion criteria:** NM	Assessment: Full length standing radiographs<br>Skeletal Maturity: NM<br>Prediction of LLD at maturity/Timing: Paley's multiplier method	Post-operative: Full weightbearing<br><br>Removal: NM	Total: 11 patients<br>Sex: 5 M/ 6 F<br>Age: 8.7 ± 2.3 yrs<br>Etiology: Hemihypertrophy/BWS<br>Bone: Femur 3, Tibia 2, Femur/Tibia 6<br>Side: NM	5 ± 2.5 yrs (18 mos–8.4 yrs)
**De Pellegrin 2021 [18]**<br>Country: Italy<br>Type of study: Case series<br>Duration: 2007-2021<br>Epiphysiodesis: TBP	**Inclusion criteria:** Beckwith-Wiedemann Syndrome patients (BWS) genetic or clinical diagnosis associated with LLD, availability of data about LLD, and height before the first surgical procedure and of the same data at the most recent follow-up.<br>**Exclusion criteria:** Genetic diagnosis that excludes BWS pattern, such as isolated hemihypertrophy, and the impossibility to collect correct measurement before and after surgery.	Assessment: Tape-measure method<br>Skeletal Maturity: NM<br>Prediction of LLD at maturity/Timing: NM	Post-operative: NM<br><br>Removal: Plates were removed when LLD was eliminated.	Total: 22 patients<br>Sex: 7 M/ 15 F<br>Age: 7.94 yrs (2.91–14.41)<br>Etiology: BWS<br>Bone: Femur 1, Tibia 18, Femur/Tibia 3<br>Side: R 14/ L 8	5 yrs and 7 mos
**Masquijo 2020 [19]**<br>Country: Argentina, Chile<br>Type of study: Retrospective observational study (Case-series)<br>Duration: 1/2014-1/2019<br>Epiphysiodesis: TBP	**Inclusion criteria:** Patients who presented with a minimal follow-up of 12 months from removal of the metaphyseal screw.<br>**Exclusion criteria:** The patients with incomplete record.	Assessment: NM<br>Skeletal Maturity: NM<br>Prediction of LLD at maturity/Timing: NM	Post-operative: NM<br><br>Removal: NM	Total: 4 patients<br>Sex: 4 M/ 0 F<br>Age: 7 yrs (4-9)<br>Etiology: Femoral hemimelia<br>Bone: Femur 1, Femur/Tibia 3<br>Side: R 3/ L 1	NM

**Sinha 2018 [20]**<br>Country: Israel<br>Type of study: Retrospective observational study (Case-series)<br>Duration: 1/2007-7/2015<br>Epiphysiodesis: TBP	**Inclusion criteria:** TBP in the proximal tibia for correction of LLD.<br>**Exclusion criteria:** Patients requiring concomitant osteotomy, or those with inadequate chart information or radiographic records.	Assessment:<br>Full length standing radiographs<br>Skeletal Maturity: NM<br>Prediction of LLD at maturity/Timing: NM	Post-operative: NM<br><br>Removal: NM	Total: 8 patients<br>Sex: NM<br>Age: NM<br>Etiology:<br>Hemihypertrophy, Idiopathic LLD<br>Bone: Tibia 8<br>Side: NM	NM
**Joeris 2017 [21]**<br>Country: Multicenter<br>Type of study: Retrospective international multicenter study<br>Duration: 10/2012-12/2013<br>Epiphysiodesis: TBP (LLD and VVD) *	**Inclusion criteria:**<br>Treatment of VVD of the knee and/or LLD because of any of the following: Diseases or syndromes affecting the growth plate (e.g. Blount's disease), Post-traumatic, Postinfectious, Idiopathic aetiology<br>**Exclusion criteria:** Any tumor, Cerebral palsy, Total epiphyseal closure	Assessment: Radiographs<br>Skeletal Maturity: NM<br>Prediction of LLD at maturity/Timing:<br>The planning and timing of surgery was based on the present discrepancy and not on the expected discrepancy at maturity.	Post-operative:<br>According to each center protocol<br><br>Removal:<br>According to each center protocol	Total: 32 patients (23 LLD, 9 LLD and VVD)<br>Sex: NM<br>Age: NM<br>Etiology: Idiopathic LLD<br>Bone: Femur 8, Tibia 4, Femur/Tibia 20<br>Side: NM	25.3 mos patients VVD and LLD, 18.9 mos LLD patients
**Gaumetou 2016 [22]**<br>Country: France<br>Type of study: Prospective observational study<br>Duration: 12/2007-7/2011<br>Epiphysiodesis: TBP	**Inclusion criteria:**<br>Patients treated by TBP for LLD. Minimum 18-month fu was required to be consistent with previous literature, but all patients were clinically and radiologically followed until skeletal maturity.<br>**Exclusion criteria:** Patients with previous surgery, angular deformities (genu valgum or varum > 5 degrees), or insufficient radiologic follow-up.	Assessment: Standing full-length low dose stereoradiograghy<br>Skeletal Maturity:<br>Greulich and Pyle atlas<br>Prediction of LLD at maturity/Timing:<br>Hechard and Carlioz table and the Paley multiplier method.	Screws inserted parallel to the growth plate and others had screws positioned divergent.<br><br>Post-operative:<br>Total weight-bearing immediately and patients were discharged on 1st postoperative day.<br><br>Removal: NM	Total: 32 patients<br>Sex: NM<br>Age: 12.5±1.6 yrs<br>Etiology: Idiopathic LLD<br>Bone: Femur 15, Tibia 25<br>Side: NM	Maturity 30±8 mos

**Jochymek 2015 [23]**<br>Country:<br>Czech Republic<br>Type of study:<br>Case series<br>Duration:<br>1/2014-1/2015<br>Epiphysiodesis: TBP	**Inclusion criteria:**<br>Patients that undergone surgery TBP<br>**Exclusion criteria:** NM	Assessment:<br>Standardized long-leg radiographs<br>Skeletal Maturity: NM<br>Prediction of LLD at maturity/Timing: NM	Overcorrection due to possible rebound<br><br>Post-operative:<br>Bearing weight with crunches first post-operative day, max for 2 weeks, rehabilitation<br><br>Removal:<br>Plates were removed when LLD was eliminated	Total: 9 patients<br>Sex: NM<br>Age: NM<br>Etiology: Post-traumatic<br>Bone: Femur 7, Tibia 2<br>Side: NM	12.7 mos (11.2–13.8 mos)
**Pendleton 2013 [24]**<br>Country: USA<br>Type of study:<br>Retrospective observational study (Case-series)<br>Duration:<br>10/2004- 12/2010<br>Epiphysiodesis: TBP	**Inclusion criteria:**<br>Patients who underwent guided growth of the femur, tibia, or both for a LLD of less than 5 cm; had adequate radiographs; had no knee or ankle contractures; had undergone no concomitant lengthening or shortening procedures; had undergone only 1 epiphysiodesis procedure (although they may have previously undergone hemiepiphysiodesis); had no significant angular deformity prior to treatment that recurred during treatment; and were followed to maturity or plate removal.<br>**Exclusion criteria:** NM	Assessment:<br>Standardized long-leg radiographs<br>Skeletal Maturity:<br>Radiograph of the hand and standardized charts for growth per year in the proximal tibia (6 mm) and distal femur (10 mm)<br>Prediction of LLD at maturity/Timing: NM	Goal was to decrease the discrepancy to a more manageable one that could be treated with shoe lift.<br><br>Post-operative: NM<br><br>Removal: NM	Total: 34 patients<br>Sex: 18 M/ 16 F<br>Age: 12.5 yrs (7-16)<br>Etiology: Congenital<br>Bone: Femur 17, Tibia 20, Femur/Tibia 7<br>Side: NM	Maturity, 28 mos (11–54 mos)

**Percutaneous epiphysiodesis using transphyseal screws (PETS)**					
**Dodwell 2017 [25]**<br>Country: USA<br>Type of study: Retrospective review - Case series<br>Duration: 2007-2014<br>Epiphysiodesis: PETS	**Inclusion criteria:**<br>Patients who underwent distal femoral and/or proximal tibial PETS, minimum 1 year follow-up.<br>**Exclusion criteria:**<br>Lost to follow-up prior to 1 year follow-up, if they underwent concomitant ipsilateral lower extremity procedures at the time of PETS, or if pre-operative imaging was performed at an outside hospital and not available for review	Assessment:<br>Plain radiographs or CT scanogram were used initially while EOS low dose biplanar radiograph has been the standard for the last 5 years.<br>Skeletal Maturity:<br>Left hand radiographs<br>Prediction of LLD at maturity/Timing:<br>Greulich and Pyle, multiplier method	Post-operative: NM<br><br>Removal: NM	Total: 82 patients<br>Sex: 51 M/ 31 F<br>Age:<br>female 12.0 yrs (SD 1.4), male 13.8 yrs (SD 1.5)<br>Etiology: Developmental (acquired such as post-traumatic, infectious, iatrogenic, or unknown)<br>Bone: Femur 21, Tibia 18, Femur/Tibia 43<br>Side: R 49/ L 33	Maturity
**Song 2015 [26]**<br>Country: Korea<br>Type of study: Retrospective observational study (Case-series)<br>Duration: NM<br>Epiphysiodesis: PETS	**Inclusion criteria:**<br>Those who were followed until skeletal maturity or screw removal and did not undergo any other bony procedures that might affect leg length.<br>**Exclusion criteria:**<br>Underwent PETS later than estimated optimal epiphysiodesis timing and those with a dislodged screw, which no longer purchased the epiphysis, during follow-up.	Assessment:<br>Standing teleradiographs<br>Skeletal Maturity: NM<br>Prediction of LLD at maturity/Timing:<br>Multiplier method	Screws cross.<br>The goal of LLD correction was LLD of within 10 mm.<br><br>Post-operative: NM<br><br>Removal:<br>After targeted LLD correction had been achieved or were left unremoved by patients' choice.	Total: 59 patients (48 before timing, 11 after)<br>Sex: 36 M/ 23 F<br>Age: male 13.7 yrs (11.3-15.0) female 11.8 yrs (10.7-12.6)<br>Etiology:<br>Congenital hemihypertrophy, posttraumatic<br>Bone: Femur 50, Tibia 19<br>Side: NM	Maturity, 3.9 yrs (2.2–7.7)

**Monier 2015 [27]**<br>Country: USA<br>Type of study:<br>Retrospective review - Case series<br>Duration: NM<br>Epiphysiodesis: PETS	**Inclusion criteria:**<br>Patients who were treated with PETS for a predicted LLD 2.5 cm at skeletal maturity.<br>**Exclusion criteria:** NM	Assessment:<br>Full length standing radiographs<br>Skeletal Maturity:<br>Radiographs left hand and wrist 'Radiographic Atlas of Skeletal Development of the Hand & Wrist'<br>Prediction of LLD at maturity/Timing:<br>Green-Anderson growth remaining method, Moseley graph method, and Paley multiplier method	Screws parallel or crossed according to surgeon preference<br><br>Post-operative: NM<br><br>Removal: NM	Total: 16 patients<br>Sex: NM<br>Age: 14 yrs (11.7-16.1)<br>Etiology: Congenital<br>Bone: Femur 1, Femur/Tibia 15<br>Side: NM	Maturity, 2 yrs (0.7–5.2)
**Ilharreborde 2012 [28]**<br>Country: France<br>Type of study:<br>Retrospective review - Case series<br>Duration: 1998-2006<br>Epiphysiodesis: PETS	**Inclusion criteria:**<br>All patients with LLD treated by PETS<br>**Exclusion criteria:**<br>Patients with associated deformity in the frontal plane (genu varum or valgum > 5°) or insufficient radiological follow-up	Assessment:<br>Full length standing radiographs<br>Skeletal Maturity:<br>Greulich and Pyle<br>Prediction of LLD at maturity/Timing: NM	Screws positioned in the lateral third of the physis in the coronal plane, and in the central third in the sagittal plane<br><br>Post-operative:<br>Full weightbearing immediately<br><br>Removal: NM	Total: 45 patients<br>Sex: 25 M/ 20 F<br>Age: 12.5 yrs (8-15)<br>Etiology:<br>Congenital fibular deficiency, Traumatic<br>Bone: Femur 11, Tibia 15, Femur/Tibia 19<br>Side: NM	65 mos (SD 11)
**Khoury 2007 [29]**<br>Country: USA<br>Type of study:<br>Retrospective review - Case series<br>Duration:<br>Epiphysiodesis: PETS	**Inclusion criteria:**<br>Underwent PETS for the correction of limb length inequality<br>**Exclusion criteria**:<br>Inadequate follow-up or concomitant procedures on the same bone (such as osteotomy)	Assessment: Radiographs<br>Skeletal Maturity: NM<br>Prediction of LLD at maturity/Timing:<br>Multiplier method	Femoral screws without crossing and cross tibial screws<br><br>Post-operative: NM<br><br>Removal:<br>No removal with the intent of growth resumption	Total: 30 patients*<br>Sex: 18 M/ 12 F<br>Age: NM<br>Etiology: Idiopathic<br>Bone: Femur 15, Tibia 6, Femur/Tibia 9<br>Side: NM	Maturity

**Nouh 2004 [30]**<br>Country: Australia<br>Type of study:<br>Prospective review - Case series<br>Duration: 1998-2002<br>Epiphysiodesis: PETS	**Inclusion criteria**:<br>Children with at least 2 years of growth remaining; follow-up greater than 1 year; patients undergoing PETS as their primary corrective procedure; LLD of 2 to 5 cm.<br>**Exclusion criteria:**<br>Lost to fu or fu < 1 yr	Assessment:<br>Full length standing radiographs<br>Skeletal Maturity: NM<br>Prediction of LLD at maturity/Timing: NM	Screws cross the physis at the junction of the central and peripheral quarters<br><br>Post-operative: NM<br><br>Removal:<br>Desired correction and the screws were removed	Total: 9 patients*<br>Sex: NM<br>Age: NM<br>Etiology:<br>Congenital, renal disease<br>Bone: NM<br>Side: NM	2.4<br>(1–4.5 yrs)
**Metaizeau 1999 [6]**<br>Country: France<br>Type of study:<br>Prospective review - Case series<br>Duration: NM<br>Epiphysiodesis: PETS	**Inclusion criteria:**<br>Patients who underwent PETS and follow up after skeletal maturity.<br>**Exclusion criteria:** NM	Assessment:<br>Scanograms<br>Skeletal Maturity: NM<br>Prediction of LLD at maturity/Timing: NM	Screws cross and parallel<br><br>Post-operative:<br>weight bearing as tolerated within 48 h of surgery<br><br>Removal: NM	Total: 32 patients*<br>Sex: NM<br>Age: 12.9 yrs<br>Etiology:<br>Post fracture limb overgrowth (PFLO)<br>Bone: NM<br>Side: NM	Maturity
**Blount Staples**					
**Gorman 2009 [3]**<br>Country: USA<br>Type of study:<br>Retrospective observational study (Case-series)<br>Duration: 1990-2005<br>Epiphysiodesis:<br>Blount Staples	**Inclusion criteria:**<br>Stapling had to have been performed on a normal lower limb or one with overgrowth due to hemihypertrophy, and a minimum of two years of follow-up.<br>**Exclusion criteria:**<br>Not complete adequate preoperative, postoperative, and final follow-up radiographs	Assessment:<br>Long standing anteroposterior lower-extremity radiographs<br>Skeletal Maturity:<br>Girls 14 yrs, Boys 16 yrs<br>Prediction of LLD at maturity/Timing: NM	Four staples (2 on each side of the knee)<br><br>Post-operative: NM<br><br>Removal: Maturity or when appropriate or maximal correction LLD	Total: 54 patients<br>Sex: 30 M/ 24 F<br>Age: 12.0 yrs<br>(7.9 - 15.1)<br>Etiology:<br>Neurologic disorders<br>Bone: Femur 15, Tibia 18, Femur/Tibia 21<br>Side: 25 R/ 29 L	Maturity

**Skytta 2003 [31]**<br>Country: Finland<br>Type of study: Retrospective observational study (Case-series)<br>Duration: 1957-1999<br>Epiphysiodesis: Blount Staples	**Inclusion criteria:** Patients who had undergone temporary epiphyseal stapling of the knee due to LLD<br>**Exclusion criteria:** Reoperations, oligoarthritis, stapling period that was too short at the evaluation time, deficient data	Assessment: NM<br>Skeletal Maturity: Radiographs (hands and knees) to verify the epiphyseal plate, state of pubertal development, and level of serum alkaline phosphatase.<br>Prediction of LLD at maturity/Timing: NM	Post-operative: NM<br><br>Removal: NM	Total: 71 patients<br>Sex: 26 M/ 45 F<br>Age: 11 yrs (5–16)<br>Etiology: Juvenile idiopathic arthritis (JIA)<br>Bone: NM<br>Side: 37 R/ 34 L	12 mos (6–59)
**Raab 2001 [32]**<br>Country: Germany<br>Type of study: Retrospective observational study (Case-series)<br>Duration: 1970-1991<br>Epiphysiodesis: Blount Staples	**Inclusion criteria:** Patients treated by Blount's epiphyseal stapling for LLD<br>**Exclusion criteria:** NM	Assessment: Full length standing radiographs<br>Skeletal Maturity: Atlas of Greulich and Pyle<br>Prediction of LLD at maturity/Timing: Anderson and Green or the Moseley chart	3 Blount staples were inserted anterior, central and posterior parallel to the physis.<br><br>Post-operative: NM<br><br>Removal: NM	Total: 24 patients<br>Sex: 9 M/ 15 F<br>Age: 9.8 yrs (4.7-14.2)<br>Etiology: Pathological shortening of the leg, as a secondary LLD (post-traumatic, post-infectious etc.)<br>Bone: Femur 5, Tibia 2, Femur/Tibia 17<br>Side: 12 R/ 12 L	4.55 yrs
**Sengupta 1993 [33]**<br>Country: India<br>Type of study: Retrospective observational study (Case-series)<br>Duration: 1964-1990<br>Epiphysiodesis: Blount Staples	**Inclusion criteria:** Patients who had epiphyseal stapling<br>**Exclusion criteria:** NM	Assessment: Clinically and whenever possible by radiography<br>Skeletal Maturity: NM<br>Prediction of LLD at maturity/Timing: NM	Two staples placed on either side of the femur.<br><br>Post-operative: Extend and flex the knee from the first day and to walk from the 3rd day.<br><br>Removal: NM	Total: 503 patients<br>Sex: NM<br>Age: NM<br>Etiology: Poliomyelitis, neonatal infections of the hip, femur and knee<br>Bone: NM<br>Side: NM	Maturity

**Watillon 1986 [34]**<br>Country: Belgium<br>Type of study: Retrospective observational study (Case-series)<br>Duration: 02/1960-07/1982<br>Epiphysiodesis: Blount Staples	**Inclusion criteria:** Surgical stapling of the epiphysis<br>**Exclusion criteria:** NM	Assessment: NM<br>Skeletal Maturity: NM<br>Prediction of LLD at maturity/Timing: NM	Three staples on each of medial and lateral surfaces<br><br>Post-operative: NM<br><br>Removal: NM	Total: 29 patients<br>Sex: 17 M/ 12 F<br>Age: 9.3-15.3 yrs<br>Etiology: Congenital, traumatic<br>Bone: Femur 19, Tibia 1, Femur/Tibia 9<br>Side: NM	Maturity
**Mukherji 1979 [35]**<br>Country: India<br>Type of study: Retrospective observational study (Case-series)<br>Duration: 1965-1973<br>Epiphysiodesis: Blount Staples	**Inclusion criteria:** Surgical stapling of the epiphysis for correction of LLD<br>**Exclusion criteria:** Excluded if cause of LLD was osteomyelitis	Assessment: NM<br>Skeletal Maturity: NM<br>Prediction of LLD at maturity/Timing: Anderson and Green	3 staples on each of medial and lateral surfaces.<br><br>Post-operative: Immobilized in posterior plaster slab for 2 wks<br><br>Removal: After correction	Total: 51 patients (47 reported for final LLD)<br>Sex: NM<br>Age: NM<br>Etiology: Poliomyelitis<br>Bone: Femur 46 Femur/Tibia 5<br>Side: NM	NM
**Cabalzar 1978 [36]**<br>Country: Switzerland<br>Type of study: Retrospective observational study (Case-series)<br>Duration: 1955-1968<br>Epiphysiodesis: Blount Staples	**Inclusion criteria:** Surgical stapling of the epiphysis<br>**Exclusion criteria:** NM	Assessment: NM<br>Skeletal Maturity: Greulich and Pyle, Tanner stages<br>Prediction of LLD at maturity/Timing: NM	Post-operative: NM<br><br>Removal: NM	Total: 78 patients<br>Sex: 49 M/ 44 F<br>Age: NM<br>Etiology: Poliomyelitis<br>Bone: NM<br>Side: NM	NM

**May 1965 [37]**<br>Country: USA<br>Type of study:<br>Retrospective observational study (Case-series)<br>Duration: 1949-1956<br>Epiphysiodesis:<br>Blount Staples	**Inclusion criteria:**<br>Surgical stapling of the epiphysis<br>**Exclusion criteria:** NM	Assessment:<br>Clinically and radiographs<br>Skeletal Maturity: NM<br>Prediction of LLD at maturity/Timing: NM	Post-operative: NM<br><br>Removal: NM	Total: 53 patients<br>Sex: NM<br>Age: NM<br>Etiology: Poliomyelitis<br>Bone: NM<br>Side: NM	NM
**PETS/TBP/Staples and Permanent Epiphysiodesis [Phemister/ Percutaneous Epiphysiodesis (PE)]**					
**Cohen 2021[38]**<br>Country: USA<br>Type of study:<br>Cost analysis<br>Duration:<br>01/2004-07/2017<br>Epiphysiodesis:<br>PE, <u>PETS</u>	**Inclusion criteria:**<br>Femoral, tibial and femoral-tibial epiphysiodesis. At least 2 yrs fu or skeletally immature at last fu<br>**Exclusion criteria:** Patients who did not have drill or screw epiphysiodesis. Epiphysiodesis of the distal tibia and/or fibula, hemiepiphysiodesis, or epiphysiodesis originally with TBP	Assessment: Radiographs<br>Skeletal Maturity: NM<br>Timing: NM<br>Prediction of LLD at maturity: NM	Post-operative: NM<br><br>Removal: NM	Total: 235 patients (155 PE, <u>80 PETS</u>)<br>Sex: 116 M/ 119 F<br>Age: 13 yrs (8.4-16.7)<br>Etiology: NM<br>Bone: NM<br>Side: NM	Maturity
**Borbas 2019 [39]**<br>Country: Switzerland<br>Type of study:<br>Case series<br>Duration: 2006-2012<br>Epiphysiodesis:<br>PE, <u>TBP</u>	**Inclusion criteria:**<br>LLD correction with epiphysiodesis and a minimum fu of 12 months after surgery<br>**Exclusion criteria:**<br>Patients with additional correction of angular deformities, skeletal dysplasia, malignancy, Blount disease, fu < 1 yr	Assessment:<br>Radiographs Long-Standing<br>Skeletal Maturity:<br>Skeletal age was analyzed according to the method of Greulich and Pyle<br>Timing: NM<br>Prediction of LLD at maturity: NM	Expected LLD at maturity had to be at least 2 cm.<br><br>Post-operative: Full weight bearing as tolerated on crutches immediately.<br><br>Removal: NM	Total: 38 patients (21 PE, <u>17 TBP</u>)<br>Sex: 24 M/14 F<br>Age: 13.6 yrs<br>Etiology: Idiopathic/ Congenital<br>Bone: Femur 16 Tibia 8 Femur/Tibia 14<br>Side: 13 R/ 25 L	578 days

**Troy 2018 [40]**<br>Country: USA<br>Type of study:<br>Case series<br>Duration: 2004-2015<br>Epiphysiodesis:<br>PE, PETS	**Inclusion criteria:**<br>All growing children and adolescents up to 18 years of age who were treated with either PETS or PE to correct a congenital or acquired LLD between 2 cm and 6 cm and returned for clinical fu at least 2 yrs postoperatively.<br>**Exclusion criteria:**<br>Patients with unverified LLD or who underwent hemiepiphysiodesis procedures.	Assessment: Scanogram<br>Skeletal Maturity: Bone age was determined by hand radiographs and the Greulich and Pyle method.<br>Timing: NM<br>Prediction of LLD at maturity:<br>Green-Anderson chart.	Post-operative:<br>Limited ambulation or weight-bearing while wearing a knee immobilizer for 2-4 weeks, then return to non-contact activity after a month.<br><br>Removal: NM	Total: 115 patients<br>(92 PE, 23 PETS)<br>Sex: 54 M/61 F<br>Age: 12.6 yrs (1.63)<br>Etiology: NM<br>Bone: Femur 61<br>Tibia 27 Femur/Tibia 27<br>Side: 55 R/ 60 L	3.7 yrs<br>(IQR 2.8–4.2)
**Bayhan 2017 [41]**<br>Country: USA<br>Type of study:<br>Case series<br>Duration: 2004-2012<br>Epiphysiodesis:<br>PE, TBP	**Inclusion criteria:**<br>All patients with an LLD between 2.5 and 5 cm who underwent either TBP or PE of the distal femur and/ or proximal tibia.<br>**Exclusion criteria:** Patients who had additional surgery or angular deformities on the ipsilateral limb at the time of the epiphysiodesis, or diagnosis of skeletal dysplasia, malignancy, or Blount disease.	Assessment: Scanogram, Radiographs Long-Standing<br>Skeletal Maturity:<br>The Greulich and Pyle atlas was used to determine the skeletal age.<br>Timing:<br>Moseley's straight-line method<br>Prediction of LLD at maturity: NM	Post-operative: NM<br><br>Removal: NM	Total: 72 patients<br>(48 PE, 24 TBP)<br>Sex: 34 M/38 F<br>Age: TBP 12 years (± 2)<br>PE 13 years (± 1.5)<br>Etiology:<br>Idiopathic, hemihypertrophy<br>Bone: Femur 46<br>Tibia 11 Femur/Tibia 15<br>Side: 40 R/ 32 L	TBP: 26 mos (± 15)
**Babu 2014 [42]**<br>Country: UK<br>Type of study:<br>Case series<br>Duration: 1999-2008<br>Epiphysiodesis:<br>PE, PETS	**Inclusion criteria:**<br>At least 1-year fu.<br>**Exclusion criteria:** LLD > 5 cm, simultaneous lengthening procedures were performed on the contralateral side; follow-up was under 1 year and if the medical notes and radiographs were incomplete.	Assessment: Scanogram, Radiographs Long-Standing<br>Skeletal Maturity: Left wrist to accurately determine bone age.<br>Timing: NM<br>Prediction of LLD at maturity:<br>Both the Moseley straight-line chart and Paley's multiplier.	Post-operative: NM<br><br>Removal: NM	Total: 40 patients<br>(14 PETS, 26 Canale- PE)<br>Sex: 23 M/17 F<br>Age: boys 13.3 yrs<br>girls 11.8 yrs<br>Etiology: Congenital<br>Bone: Femur 20<br>Tibia 8 Femur/Tibia 12<br>Side: 24 R/ 16 L	2.2 yrs<br>(12-72 mos)

**Stewart 2013 [43]**<br>Country: USA<br>Type of study:<br>Case series<br>Duration:<br>01/2003-08/2009<br>Epiphysiodesis:<br>PE, TBP	**Inclusion criteria:**<br>Patients received an epiphysiodesis of the distal femur and/or proximal tibia for LLD.<br>**Exclusion criteria:** Inadequate medical records or radiographic fu to determine success or failure of treatment. There were several patients who were treated using both drill epiphysiodesis and 8-plates; they were also excluded.	Assessment:<br>Radiographs Long-Standing<br>Skeletal Maturity: NM<br>Timing: NM<br>Prediction of LLD at maturity: NM	Post-operative: NM<br><br>Removal: NM	Total: 27 patients<br>(16 PE, 11 TBP)<br>Sex: 11 M/16 F<br>Age: TBP: 13 yrs<br>PE: 12.5 yrs<br>Etiology: NM<br>Bone: NM<br>Side: 12 R/ 15 L	TBP:<br>592.6 days
**Campens 2010 [44]**<br>Country: Belgium<br>Type of study:<br>Case series<br>Duration: 1987-2008<br>Epiphysiodesis:<br>Phemister, PETS, PE	**Inclusion criteria:**<br>Patients followed for LLD were treated by surgical epiphysiodesis.<br>**Exclusion criteria:** Underwent a contralateral leg lengthening or because of lack of data. Blount stapling technique.	Assessment:<br>Orthoroentgenogram, teleroentgenogram<br>Skeletal Maturity:<br>Skeletal age Greulich and Pyle atlas, Sauvegrain's method. The growth of the lower limb was considered arrested at 15 yrs in girls and 17 yrs in boys, or when the Risser staging was 4.<br>Timing: NM<br>Prediction of LLD at maturity:<br>Green and Anderson.	Post-operative: NM<br><br>Removal: NM	Total: 80 patients<br>(33 Phemister, 34 PE<br>15 PETS)<br>Sex: 50 M/30 F<br>Age: 13.4 yrs (9.3-16.3)<br>Etiology: Idiopathic, malformation<br>Bone: Femur 42<br>Tibia 2<br>Femur/Tibia 20<br>Femur/Tibia/Fibula 14<br>Tibia/Fibula 4<br>Side: 43 R/ 37 L	Maturity
**Frediani 1987 [45]**<br>Country: Italy<br>Type of study:<br>Case series<br>Duration: 1978-1985<br>Epiphysiodesis:<br>Phemister,<br>Blount staples	**Inclusion criteria:**<br>Epiphysiodesis for LLD **Exclusion criteria:** NM	Assessment:<br>Teleradiographs<br>Skeletal Maturity:<br>Left wrist to accurately determine bone age.<br>Timing: NM<br>Prediction of LLD at maturity:<br>Green–Anderson growth-remaining chart and Moseley graphs.	Blount: 3 staples each side.<br><br>Post-operative:<br>4 wks of cast immobilization<br><br>Removal: NM	Total: 16 patients<br>(2 Blount staples/<br>14 Phemister)<br>Sex: NM<br>Age: 11.5 yrs (8-14)<br>Etiology:<br>Congenital, Idiopathic<br>Bone: Femur 9<br>Tibia 7<br>Side: NM	NM

PETS/TBP/Staples					
**Younis 2022 [46]**<br>Country: USA<br>Type of study: Retrospective observational study (Case-series)<br>Duration: 06/2008-01/2019<br>Epiphysiodesis: TBP, PETS	**Inclusion criteria:** Patients who had open physes at the time of surgery and received TBP or PETS of the distal femur and/or proximal tibia for the treatment of LLD<br>**Exclusion criteria:** NM	Assessment: Lower extremity scanograms and anteroposterior standing full-length lower extremity radiographs<br>Skeletal Maturity: 14 yrs girls and 16 yrs in boys<br>Prediction of LLD at maturity/Timing: Paley multiplier method	Single plate was used for TBP on each side of the physis in distal femur/proximal tibia. PETS was carried out with either parallel or crossing screws<br><br>Post-operative: Physical therapy when needed<br><br>Removal: NM	Total: 27 patients (14 TBP, 13 PETS)<br>Sex: 13 M/14 F<br>Age: 12.0 ± 1.9 yrs<br>Etiology: Congenital/ Trauma<br>Bone: Femur 6, Tibia 11, Femur/Tibia 15<br>Side: 20 R/ 12 L	TBP: 5.4 ± 2.9 yrs<br><br>PETS: 2.5 ± 1.1 yrs
**Vogt 2021 [47]**<br>Country: Germany<br>Type of study: Prospective study and use of retrospective data<br>Duration: 2014-2019 (prospective), 1970-2014 (retrospective)<br>Epiphysiodesis: Blount Staples, TBP, Rigid staples (RigidTack)	**Inclusion criteria:** Children and adolescents who showed adequate residual growth potential for equalization or reduction of a predicted LLD of ≥ 2 cm at skeletal maturity.<br>**Exclusion criteria:** Patients with a predicted LLD of < 2 cm at skeletal maturity	Assessment: Radiographs<br>Skeletal Maturity: NM<br>Prediction of LLD at maturity/Timing: Multiplier method	Post-operative: NM<br><br>Removal: Removal of the rigid staples was performed at skeletal maturity or at the time of LLD equalization	Total: 220 patients (45 Rigid staples, 38 TBP, 137 Blount staples)<br>Sex: 143 M/88 F<br>Age: Rigid: 12.1 yrs (8-14) TBP: 12.1 yrs (10-16) Blount: 11.8 yrs (6-16)<br>Etiology: Idiopathic, congenital<br>Bone: Femur 68, Tibia 38, Femur/Tibia 136<br>Side: 117 R/ 103 L, Bilateral 11	Rigid staples 24.4 mos (8–49), TBP 36.0 mos (7–83), Blount staples 27.8 mos (6–94)

**Cheng 2021 [8]**<br>Country: Taiwan<br>Type of study: Retrospective observational study (Case-series)<br>Duration: 2010-2017<br>Epiphysiodesis: TBP, PETS	**Inclusion criteria:**<br>Patients with LLD of 2-5 cm with adequate growth remaining, who underwent temporary epiphysiodesis TBP or PETS<br>**Exclusion criteria:**<br>Patients who had concurrent osteotomy/epiphysiodesis at the ipsilateral proximal femur and had incomplete radiographic data	Assessment: Scanogram<br>Skeletal Maturity: NM<br>Prediction of LLD at maturity/Timing: NM	PETS: screws crossing<br>TBP: 2 screws were then fixed at a divergent position<br><br>Post-operative: NM<br><br>Removal: NM	Total: 53 patients (38 TBP, 15 PETS)<br>Sex: 25 M/28 F<br>Age: 11.4 yrs (6-14)<br>Etiology: Cerebral palsy, idiopathic<br>Bone: Femur 17, Tibia 16, Femur/Tibia 20<br>Side: NM	25.2 mos (24–31)
**Frommer 2021 [48]**<br>Country: Germany<br>Type of study: Retrospective observational study (Case-series)<br>Duration: 2009-2020<br>Epiphysiodesis: Staples (RigidTack and FlexTack), TBP (eight-plate and PediPlate)	**Inclusion criteria:**<br>Temporary tibial epiphysiodesis with or without concomitant femur epiphysiodesis at an age 7-16 yrs for correction of a predicted LLD 2-5 cm at maturity<br>**Exclusion criteria:**<br>Patients with permanent epiphysiodesis, history of previous surgery of the longer leg or a mechanical axis deviation (MAD) ≥ 2.5 cm, as well as patients who received systemic growth-affecting treatment such as hormone- or chemotherapy	Assessment: Long standing anteroposterior radiographs<br>Skeletal Maturity: NM<br>Prediction of LLD at maturity/Timing: Multiplier Method for congenital etiologies and the Moseley Straight-Line Graph for developmental or acquired etiologies	Post-operative: Immediate full weight bearing<br><br>Removal: After equalization of LLD or closing of the growth plate	Total: 58 patients (31 TBP,6 PediPlate, 5 FlexTack, 16 RigidRack)<br>Sex: 39 M/19 F<br>Age: 12.2 yrs (7-15)<br>Etiology: Idiopathic, hemihypertrophy, posttraumatic LLD<br>Bone: Tibia 7, Femur/Tibia 51 (27 with fibula)<br>Side: NM	36.2 mos (14–78)
**Lee 2018 [49]**<br>Country: Taiwan<br>Type of study: Retrospective observational study (Case-series)<br>Duration: 2008-2012<br>Epiphysiodesis: TBP, Blount Staples	**Inclusion criteria:**<br>Children with LLD who were treated by temporary epiphysiodesis using plates or staples, between the ages of 10-14 years and had more than 2 years of follow-up.<br>**Exclusion criteria:**<br>Children who had LLD from skeletal dysplasia or metabolic diseases	Assessment: Scanogram<br>Skeletal Maturity: NM<br>Prediction of LLD at maturity/Timing: NM	One to two staples were used at the medial and lateral sides of a physis.<br><br>Post-operative: NM<br><br>Removal: NM	Total: 19 patients (10 staples, 9 TBP)<br>Sex: 6 M/13 F<br>Age: 12.1 yrs (10.0-13.8)<br>Etiology: Idiopathic LLD<br>Bone: Femur 8, Tibia 3, Femur/Tibia 8<br>Side: NM	4.1 yrs

**Corradin 2017 [50]**<br>Country: Italy<br>Type of study: Retrospective observational study (Case-series)<br>Duration: 2006-2016<br>Epiphysiodesis: TBP, Blount Staples	**Inclusion criteria:**<br>Patients diagnosed with cerebral palsy and presenting a LLD greater than 2.5 cm, only skeletally immature patients treated with epiphysiodesis on the uninvolved leg<br>**Exclusion criteria:** NM	Assessment:<br>Full length standing radiographs<br>Skeletal Maturity:<br>Patients had to have open triradiate cartilage to be skeletally immature<br>Prediction of LLD at maturity/Timing:<br>Green–Anderson growth-remaining	Post-operative:<br>Below-knee cast for 5–6 wks<br><br>Removal:<br>Skeletal maturity and/or LLD balanced	Total: 10 patients<br>Sex: 7 M/3 F<br>Age: 12.7 yrs (10–16)<br>Etiology: Cerebral Palsy<br>Bone: NM<br>Side: 6 R/ 4 L	6.7 yrs (3–10) [Maturity]
**Siedhoff 2014 [51]**<br>Country: Germany<br>Type of study: Retrospective observational study (Case-series)<br>Duration: NM<br>Epiphysiodesis: TBP, Blount Staples	**Inclusion criteria:**<br>(1) a temporary epiphysiodesis performed for LLD of up to 5 cm (predicted LLD at time of skeletal maturity), (2) consistent preoperative, postoperative, and follow-up radiographs, and (3) skeletal maturity at the time of final follow-up examination<br>**Exclusion criteria:** NM	Assessment:<br>Radiographs Long-Standing<br>Skeletal Maturity: NM<br>Prediction of LLD at maturity/Timing:<br>Anderson and Green growth remaining charts and the Paley multiplier method	2 Blount staples or one TBP on the medial and lateral side of the physis, parallel to the physis<br><br>Post-operative: NM<br><br>Removal:<br>Maturity or LLD balanced	Total: 34 patients, (30 Staples, 4 TBP)<br>Sex: 21 M/13 F<br>Age: 12.8 yrs (10–16)<br>Etiology: Idiopathic LLD<br>Bone: Femur 14, Tibia 8, Femur/Tibia 12<br>Side: NM	Maturity
**Lykissas 2013 [7]**<br>Country: USA<br>Type of study: Retrospective observational study (Case-series)<br>Duration: 2003-2010<br>Epiphysiodesis: TBP, Blount Staples, PETS	**Inclusion criteria:**<br>(a) LLD treated with knee epiphyseal stapling, plating, or PETS; (b) concurrent epiphysiodesis of the distal femur and the proximal tibia; (c) adequate clinical and radiographic fu until skeletal maturity or minimum of 2 yrs after implant removal; (d) epiphyseal stapling, plating, or PETS as the primary procedure; and (e) absence of any other bony procedures in the lower extremities<br>**Exclusion criteria:** NM	Assessment: Scanogram<br>Skeletal Maturity:<br>Anderson and Green growth remaining chart.<br>14 yrs girls and 16 yrs in boys.<br>Assessment of skeletal age Greulich and Pyle atlas<br>Prediction of LLD at maturity/Timing:<br>Moseley straight-line graph	TBP<br>1 on each side,<br>Staple<br>3 on each side<br><br>Post-operative: NM<br><br>Removal: NM	Total: 39 patients (9 TBP, 8 staples, 22 PETS)<br>Sex: 19 M/20 F<br>Age: 12.6 yrs (8.3– 15.3)<br>Etiology: Idiopathic LLD<br>Bone: NM<br>Side: 16 R/ 23 L	NM

NM: Not mentioned, LLD: leg-length discrepancy, M: Male, F: Female, R: Right, L: Left, CDH: Congenital dislocation of the hip, fu: follow-up, TBP: Tension band plate, wks: weeks, mos: months, yrs: years. *In the analysis we included only the LLD patients' information.

Quality Assessment – Table S4

FOR NON-RANDOMIZED STUDIES (MINORS)

The items are scored 0 (not reported), 1 (reported but inadequate) or 2 (reported and adequate). The global ideal score is 16 for non-comparative studies and 24 for comparative studies.

MINORS—General part

1. ***A clearly stated aim:*** the question addressed should be precise and relevant in the light of available literature.
2. ***Inclusion of consecutive patients**:* all patients potentially fit for inclusion (satisfying the criteria for inclusion) have been included in the study during the study period (no exclusion or details about the reasons for exclusion).
3. ***Prospective collection of data**:* data were collected according to a protocol established before the beginning of the study.
4. ***Endpoints appropriate to the aim of the study*:** unambiguous explanation of the criteria used to evaluate the main outcome, which should be in accordance with the question addressed by the study. Also, the endpoints should be assessed on an intention-to-treat basis.
5. ***Unbiased assessment of the study endpoint**:* blind evaluation of objective endpoints and double-blind evaluation of subjective endpoints. Otherwise, the reasons for not blinding should be stated.
6. ***Follow-up period appropriate to the aim of the study**:* the follow-up should be sufficiently long to allow the assessment of the main endpoint and possible adverse events.
7. ***Loss to follow-up less than 5%:*** all patients should be included in the follow-up. Otherwise, the proportion lost to follow-up should not exceed the proportion experiencing the major endpoint.
8. ***Prospective calculation of the study size:*** information on the size of detectable difference of interest with a calculation of 95% confidence interval, according to the expected incidence of the outcome event, and information about the level for statistical significance and estimates of power when comparing the outcomes.

Additional criteria in the case of comparative study

9. ***An adequate control group:*** having a gold standard diagnostic test or therapeutic intervention recognized as the optimal intervention according to the available published data.
10. ***Contemporary groups:*** control and studied group should be managed during the same time period (no historical comparison).
11. ***Baseline equivalence of groups:*** the groups should be similar regarding the criteria other than the studied endpoints. Absence of confounding factors that could bias the interpretation of the results.
12. ***Adequate statistical analyses:*** whether the statistics were in accordance with the type of study with calculation of confidence intervals or relative risk.

Table S4 (a). NON-RANDOMIZED STUDIES WITHOUT COMPARISON

Study	Aim	Patients	Collection of data	Endpoints	Unbiased assessment	FU	Lost to FU	Study size	Sum
**Tension-Band Plates (TBP) studies**									
Tolk 2022	2	2	0	2	0	2	2	0	10
Stevens 2022	2	2	0	2	0	2	2	0	10
Petrova 2022	2	1	0	1	0	0	0	0	4
Demirel 2022	2	2	0	2	0	2	1	0	9
Erdal 2022	2	2	0	2	0	2	1	0	9
De Pellegrin 2021	1	2	0	2	0	2	2	0	9
Ozdemir 2021	2	2	0	2	0	2	2	0	10
Masquijo 2020	1	1	0	0	0	0	1	0	3
Sinha 2018	2	2	0	1	0	0	0	0	5
Joeris 2017	1	1	0	2	0	1	2	0	7
Gaumetou 2016	2	2	1	2	0	2	2	0	11
Jochymek 2015	2	2	0	1	0	1	2	0	8
Pendleton 2013	2	2	0	2	0	2	2	0	10
**Percutaneous epiphysiodesis using transphyseal screws (PETS) studies**									
Dodwell 2017	2	2	0	2	0	2	2	0	10
Song 2015	2	2	0	2	0	1	2	0	9
Monier 2015	1	2	0	2	0	1	2	0	8
Ilharreborde 2012	2	2	0	2	0	1	2	0	9
Khoury 2007	1	2	1	2	0	2	2	0	9
Nouh 2004	2	2	1	1	0	2	2	0	10
Metaizeau 1999	2	2	1	2	0	2	2	0	11

**Blount Staples studies**									
Gorman 2009	2	2	0	2	0	2	2	0	10
Skytta 2003	2	2	0	2	0	1	2	0	9
Raab 2001	1	2	0	2	0	2	2	0	9
Sengupta 1993	1	1	0	1	0	2	1	0	6
Watillon 1986	1	1	0	2	0	2	2	0	8
Mukherji 1979	1	2	0	2	0	1	2	0	8
Cabalzar 1978	1	1	0	2	0	0	0	0	4
May 1965	1	1	0	2	0	0	0	0	4
**TBP and Staples studies**									
Frommer 2021	2	1	0	2	0	1	2	0	8
Corradin 2017	2	1	0	2	0	2	2	0	9
Siedhoff 2014	2	1	0	2	0	2	2	0	9
**Blount staples and Phemister**									
Frediani 1987	1	1	0	1	0	0	1	0	4

Table S4 (b). NON-RANDOMIZED STUDIES WITH COMPARISON

Study	Aim	Patients	Collection of data	Endpoints	Unbiased assessment	FU	Lost to FU	Study size	Control	Groups	Equivalence	Statistical analysis	Sum
**PETS/TBP/Staples and Permanent Epiphysiodesis [Phemister/ Percutaneous Epiphysiodesis (PE)]**													
Cohen 2021	2	2	0	1	0	2	1	0	1	1	1	1	12
Borbas 2019	2	2	0	1	0	1	2	0	1	2	2	1	14
Troy 2018	2	1	0	2	0	1	2	0	1	2	2	1	14
Bayhan 2017	2	1	0	1	0	1	2	0	1	2	2	2	14
Babu 2014	2	1	0	1	0	1	2	0	1	2	1	1	12
Stewart 2013	2	1	0	1	0	1	2	0	1	2	2	1	13
Campens 2010	2	2	0	2	0	2	2	0	1	2	2	1	16
**PETS/TBP/Staples**													
Younis 2022	2	2	0	2	0	2	2	0	1	2	2	2	17
Cheng 2021	2	2	0	2	0	1	2	0	1	2	2	2	16
Vogt 2021	2	2	1	2	0	1	2	0	1	0	1	1	12
Lee 2018	2	2	0	2	0	2	2	0	1	1	2	2	16
Lykissas 2013	2	2	0	2	0	2	2	0	1	2	2	2	17

Table S5 (a). Other outcome measurements

Study	Number of Patients	Main outcome measurement	Results
**Tension-band plates (TBP)**			
Tolk 2022	34	Intra-articular morphology	Measures of intra-articular morphology showed a significant change only in the proximal tibial epiphysiodesis group, whereas no important changes were observed in those undergoing distal femoral epiphysiodesis.
Petrova 2022	94	Efficiency (Efficacy)	The maximum efficiency was observed with epiphysiodesis of the femur in children of the younger age group (a change of 7.59% in the length of the bone),<br>the minimum was observed with epiphysiodesis of the tibia in children of the older age group (a change in length of 2.04%).
Erdal 2022	26	ISA (inter-screw angle ISA was defined as the angle between screws on each plate)	ISA measurements showed no significant difference between medial and lateral plates at early postoperative and implant removal time.
Ozdemir 2021	11	Efficacy	6 months to 18 months, the mean efficacy of the epiphysiodesis of femurs was 27 ± 19 % (range, 0–56%) and tibias was 15 ± 19 % (range, 0–50%).<br>Age and physeal coverage rate by the screws could be factors related to the efficacy of TBP.
Gaumetou 2016	32	Efficacy	Femur: 68%, Tibial:42%
**Percutaneous epiphysiodesis using transphyseal screws (PETS)**			
Dodwell 2017	82	Efficacy	The mean efficacy for the distal femur was 97% (SD = 46%), for the proximal tibia was 108% (SD = 66%), and was 103% (SD = 57%) overall
Song 2015	48	Efficacy	Mean LLD correction efficacy was 75.5% (range, 5.0% to 114.0%) at the distal femur and 78.9% (range, 11.0% to 111.0%) at the proximal tibia.
Ilharreborde 2012	45	Efficacy	66% (SD 8) femoral, 66% (SD 9) tibial
Metaizeau 1999	32	Growth reduced	Epiphysiodesis had retarded the growth of the distal femoral physis by 79% and reduced total femoral growth to 45% of normal, and the growth of the proximal tibial physis by 86% and total tibial growth to 52% of normal at maturity.

Table S5 (b). Other outcome measurements

PETS/TBP/Staples and Permanent Epiphysiodesis [Phemister/ Percutaneous Epiphysiodesis (PE)]			
Lee 2018	19 (10 staples 9 TBP)	LLD ratio	The change of length ratio was significantly greater in the stapling group at two years. (-3.6% by stapling versus -1.8% by plating, $p < 0.05$)
Lykissas 2013	39 (9 TBP 8 staples 22 PETS)	Rate of correction	Rate of correction: TBP 1.11 cm/year, Staples 1.22 cm/year, PETS 0.59 cm/year
Stewart 2013	27 (11 TBP 16 PE)	Median improvement	Median improvement: PE:15.5 mm, TBP:4.0 mm. This difference was highly significant (P =< 0.001)
Borbas 2019	38 (21 PE 17 TBP)	MAD (mechanical axis deviation), axial devation difference	In both groups, LLD was successfully reduced after 12 and 24 months ($p < 0.001$). No difference could be seen in axial alignment and MAD change
Younis 2022	27 (14 TBP 13 PETS)	Efficacy, rate of correction	Efficacy: TBP 59%, PETS 64% at maturity. At the most recent follow-up, the efficacy of both operations was equal. Rate of correction for limbs treated with TBP was 0.49 ± 0.9 cm/ year and for limbs treated with PETS was 1.0 ± 1.1 cm/year ($p = 0.185$)
Troy 2018	115 (23 PETS 92 PE)	Percentage correction LLD	Percentage correction LLD (%): PETS 65.3% (50.7 to 80), PE 65.2% (58.4 to 71.9)
Bayhan 2017	72 (24 TBP 48 PE)	Percentage of improvement	Percentage of improvement was significantly higher in the PE group (58%) than in the TBP (41%, P= 0.031)
Cohen 2021	235 (155 PE, 80 PETS)	Cost Analysis	PE was more cost-effective due to reduced costs at each node regardless of minimal differences in complications

Table S6. Studies not defining which type of epiphysiodesis was used

Study	Patients	Epiphysiodesis Types	Outcome	Complications
Corradin 2018	10	TBP or Blount staples	NM	No complications
Frediani 1987	16	Blount or Phemister	Pre-Tibia: 3.9 cm / Post-Tibia: 0.9 cm<br>Pre-Femur: 5.3 cm/ Post-Femur: 3.1 cm	NM (low complications)

Table S7. Studies including TBP, PETS and/or staples as individual groups

Study	Number of Patients	Compared Techniques	Results
Lykissas 2013	39 patients	9 TBP<br>8 staples<br>22 PETS	A statistically significant difference was noticed in the rate of limb shortening between the stapling and PETS groups, with the former having a better rate of limb length discrepancy correction ($P = 0.045$). All the other comparisons non-significant.
Lee 2018	19 patients	10 staples<br>9 TBP	The change of length ratio was significantly greater in the stapling group at two years (-3.6% by stapling versus -1.8% by plating, $p < 0.05$).<br>This retrospective review showed longer latency and lower efficacy in decreasing LLD on using tension band plating. Stapling offered greater reduction of bone length and a quicker response to achieve a significant change.
Younis 2022	27 patients	14 TBP<br>13 PETS	Both PETS and TBP are effective at controlling LLD in our sample at the final follow-up. PETS patients, on the other hand, had a shorter OR time, a shorter hospital stay, an earlier return to activity, and a lower complication rate.
Cheng 2021	53 patients	38 TBP<br>15 PETS	At two years postoperatively, TBP and PETS caused a significant decrease in LLD ($p < 0.05$). A tendency for varus and valgus changes of the knee was found in the TBP and PETS groups, respectively.
Siedhoff 2014	34 patients	30 staples<br>4 TBP	No statistical comparison
Vogt 2021	220 patients	45 Rigid staples<br>38 TBP<br>137 Blount staples	No statistical comparison

Table S8 (a). Complications for each study according to Black [10]

	Acute complications					Long term complications					Overall	Patients
**Studies**	**I**	**II**	**IIIA**	**IIIB**	**Total**	**I**	**II**	**IIIA**	**IIIB**	**Total**		
**Tension-band plates (TBP)**												
Tolk 2022	14				14	5	1			6	20	34
Stevens 2022					0			28	11	39	39	66
Petrova 2022	-	-	-	-	-	-	-	-	-	-	-	-
Demirel 2022	1				1			9		9	10	11
Erdal 2022	1				1			3		3	4	26
De Pellegrin 2021					0		6	2		8	8	22
Ozdemir 2021					0				3	3	3	11
Masquijo 2020					0		2		1	3	3	4
Sinha 2018					0		2		1	3	3	8
Joeris 2017*					0			11		11	11	32
Gaumetou 2016	8	1			9		1	3		4	13	32
Jochymek 2015					0			2		2	2	9
Pendleton 2013	1				1			2		2	3	34
Younis 2022	3				3		2	19	4	25	28	14
Cheng 2021	6				6		1		6	7	13	38
Vogt 2021	1				1		4	20	13	37	38	38
Borbas 2019		3			3			1	1	2	5	17
Lee 2018					0			2	1	3	3	9
Bayhan 2017	8				8		3	4	2	9	17	24
Siedhoff 2014		2			2					0	2	4
Lykissas 2013	2				2			12		12	14	9
Steward 2013					0					0	0	11
**Total**	**45**	**6**	**0**	**0**	**51**	**5**	**22**	**118**	**43**	**188**	**239**	**453**

*Not clearly defined for only LLD patients, only not successful cases included.

Table S8 (b). Complications for each study according to Black [10]

	Acute complications					Long term complications					Overall	Patients
**Studies**	**I**	**II**	**IIIA**	**IIIB**	**Total**	**I**	**II**	**IIIA**	**IIIB**	**Total**		
**PETS**												
Dodwell 2017	20				20		13	6	4	23	43	82
Song 2015					0	5	3	4	3	15	15	59
Monier 2015					0		6	1		7	7	16
Ilharreborde 2012					0	5	35	24	9	73	73	45
Khoury 2007					0		7		1	8	8	30
Nouh 2004		1			1			5		5	6	9
Metaizeau 1999	7				7			7		7	14	32
Younis 2022	2				2			9		9	11	13
Cheng 2021					0		1			1	1	15
Cohen 2021					-						9 ND	80
Troy 2018	6				6			3		3	9	23
Babu 2014	2				2		1	1		2	4	14
Lykissas 2013	6				6		1	10	1	12	18	22
Campens 2010					0		1	4		5	5	15
**Total**	**43**	**1**	**0**	**0**	**44**	**10**	**68**	**74**	**18**	**170**	**223**	**455**

Table S8 (c). Complications for each study according to Black [10]

	Acute complications					Long term complications					Overall	Patients
**Studies**	**I**	**II**	**IIIA**	**IIIB**	**Total**	**I**	**II**	**IIIA**	**IIIB**	**Total**		
**Staples**												
Gorman 2009	12				12	5		20	8	33	45	54
Skytta 2003	1				1		5		1	6	7	71
Raab 2001	12				12		27	10		37	49	24
Sengupta 1993	55	9			64			15	8	23	87	503
Watillon 1986	5				5			9		9	14	29
Mukherji 1979	2				2		4	14	9	27	29	51
Cabalzar 1978					-			52	53	105	126 (21 ND)	78
May 1965	2				2		23	2	12	37	39	53
Vogt 2021	8				8		20	25	47	92	100	137
Lee 2018					0				2	2	2	10
Siedhoff 2014					0	1	3	3	3	10	10	30
Lykissas 2013	4				4			4		4	8	8
**Total**	**101**	**9**	**0**	**0**	**110**	**6**	**82**	**154**	**143**	**385**	**516**	**1048**
**RIGID staples**												
Vogt 2021 (RigidTack)	2				2		4	22	5	31	33 (11)	45
Frommer 2021 [Staples (RigidTack and FlexTack), TBP (eight-plate and PediPlate)]	10				10				8	8	18	58

Table S9. Sensitivity analysis for success rate.

Sensitivity analysis	Number of studies	Percentage	95% CI	I^2
**TBP**				
Primary Analysis	10	67.0%	54.1-78.9	69.0%
Excluding the studies with Not Mentioned success definition	9	66.0%	52.1-78.9	72.5%
**PETS**				
Primary Analysis	9	76.1%	61.0-88.7	84.5%
Excluding the studies with Not Mentioned success definition	7	68.5%	53.0-82.2	75.5%
**Staples**				
Primary Analysis	8	51.4%	37.5-65.1	85.2%
Only < 1 cm	6	53.6%	36.1-70.6	88.6%

Figure S1. (a) Forest plot for TBP including < 2cm as acceptable for successful result.

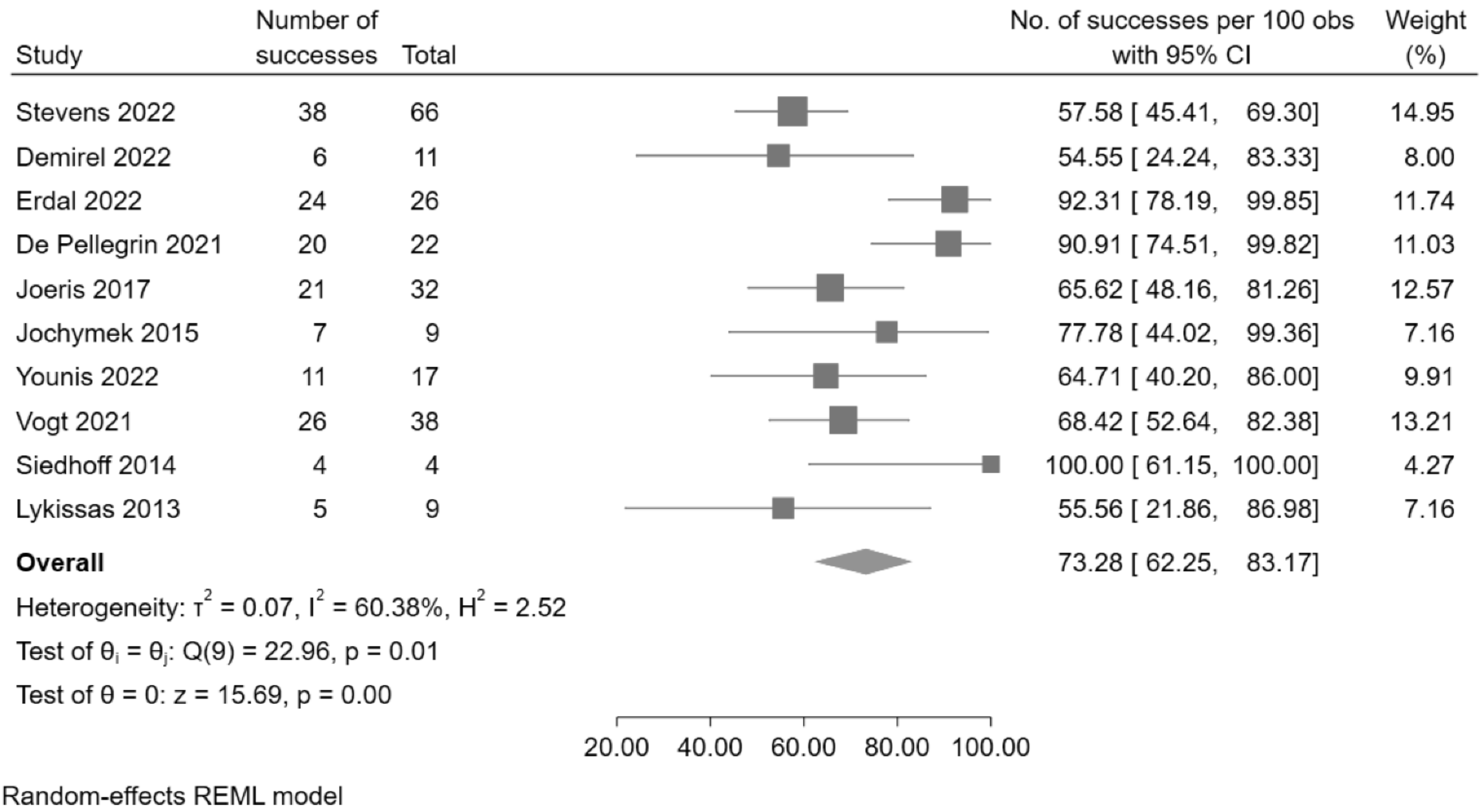

Figure S1. (b) Forest plot for PETS including < 2cm as acceptable for successful result.

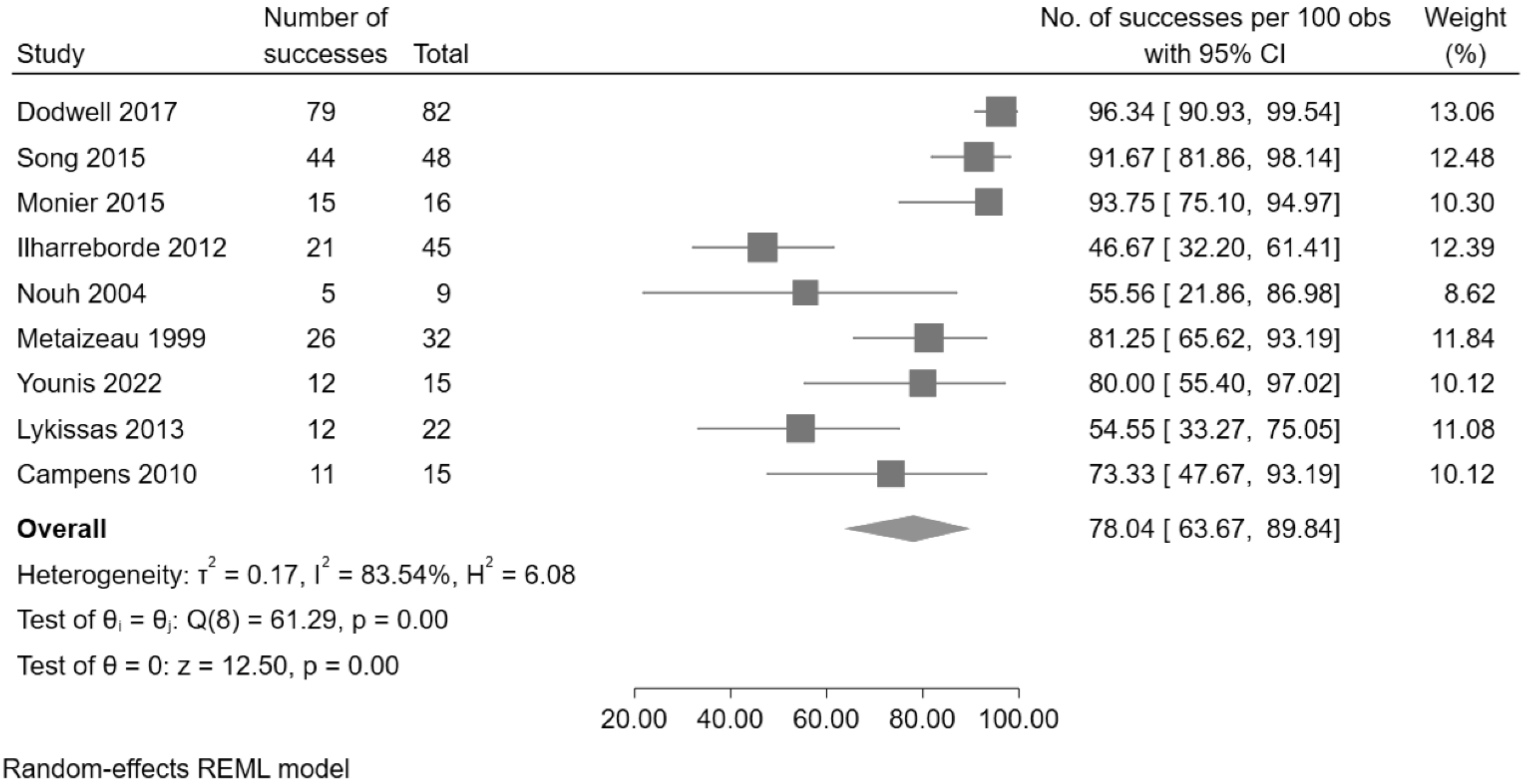

Figure S1. (c) Forest plot for Blount staples including < 2cm as acceptable for successful result.

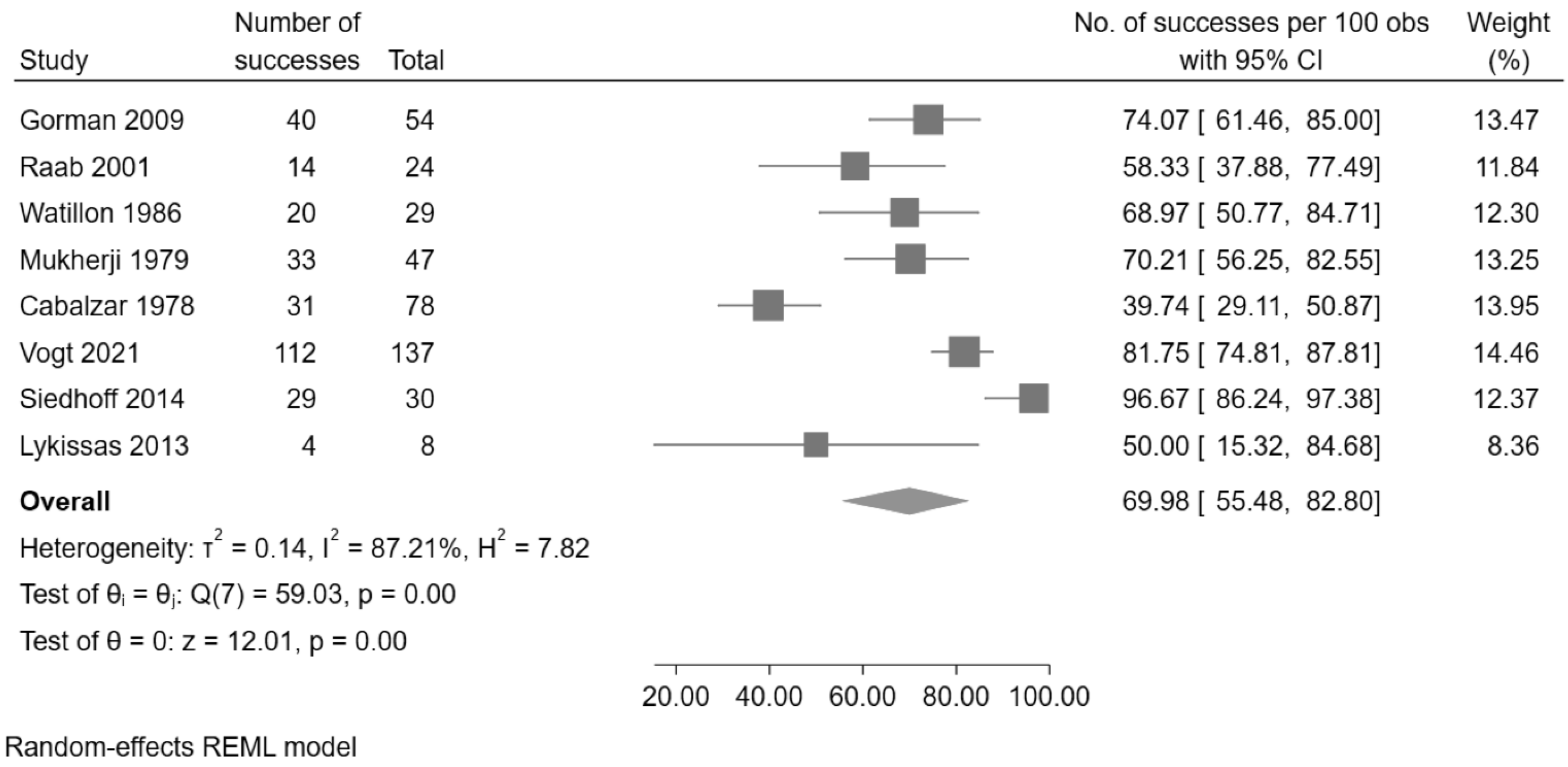

Supplement: Supplementary file 1 [file ActaO-95-41104-s1.pdf]
